# Coordinated changes in cellular behavior ensure the lifelong maintenance of the hippocampal stem cell population

**DOI:** 10.1016/j.stem.2021.01.003

**Published:** 2021-05-06

**Authors:** Lachlan Harris, Piero Rigo, Thomas Stiehl, Zachary B. Gaber, Sophie H.L. Austin, Maria del Mar Masdeu, Amelia Edwards, Noelia Urbán, Anna Marciniak-Czochra, François Guillemot

**Affiliations:** 1 Neural Stem Cell Biology Laboratory, The Francis Crick Institute, London NW1 1AT, UK; 2 Institute of Applied Mathematics, Heidelberg University, 69120 Heidelberg, Germany; 3 Interdisciplinary Center for Scientific Computing (IWR), Heidelberg University, 69120 Heidelberg, Germany; 4 Bioquant Center, Heidelberg University, 69120 Heidelberg, Germany; 5 Advanced Sequencing Facility, The Francis Crick Institute, London NW1 1AT, UK

**Keywords:** neural stem cell, hippocampus, quiescence, age, neurogenesis, dormant, resting, Ascl1, Huwe1

## Abstract

Neural stem cell numbers fall rapidly in the hippocampus of juvenile mice but stabilize during adulthood, ensuring lifelong hippocampal neurogenesis. We show that this stabilization of stem cell numbers in young adults is the result of coordinated changes in stem cell behavior. Although proliferating neural stem cells in juveniles differentiate rapidly, they increasingly return to a resting state of shallow quiescence and progress through additional self-renewing divisions in adulthood. Single-cell transcriptomics, modeling, and label retention analyses indicate that resting cells have a higher activation rate and greater contribution to neurogenesis than dormant cells, which have not left quiescence. These changes in stem cell behavior result from a progressive reduction in expression of the pro-activation protein ASCL1 because of increased post-translational degradation. These cellular mechanisms help reconcile current contradictory models of hippocampal neural stem cell (NSC) dynamics and may contribute to the different rates of decline of hippocampal neurogenesis in mammalian species, including humans.

## Highlights

• More proliferating hippocampal stem cells return to shallow quiescence with age
• Dormant stem cells enter deeper quiescence with age
• These changes drive the transition from developmental to adult neurogenesis
• Increasing degradation of ASCL1 protein by HUWE1 coordinates these changes

## Graphical abstract

**Figure:**
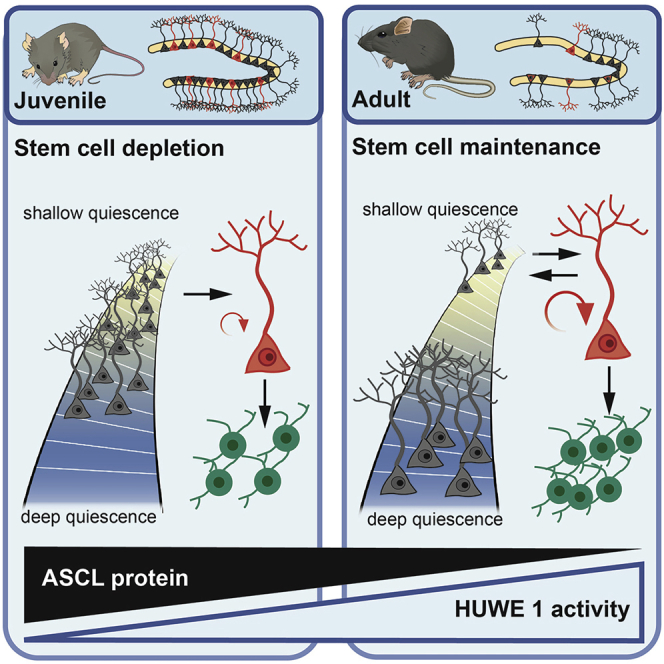

## Teaser

Harris et al. show that multiple cellular changes work in concert during early life to preserve the hippocampal stem cell population throughout adulthood in mice. In particular, more proliferating stem cells return to quiescence instead of differentiating. The changes are coordinated by increasing degradation of the pro-activation factor ASCL1.

## Introduction

Neural stem cells (NSCs) persist in restricted brain areas during adulthood, mostly in the dentate gyrus (DG) of the hippocampus and the ventricular-subventricular zone (V-SVZ) of the lateral ventricles. In mice, proliferative DG precursor cells enter quiescence during the second week after birth. By the end of the second postnatal week, quiescent DG precursors have acquired the elongated and branched morphology and arrangement in a single cell layer that typify adult NSCs (Berg et al., 2019; Hochgerner et al., 2018; Li et al., 2013; Nicola et al., 2015; Noguchi et al., 2019). There is therefore a clear histological transition between developmental neurogenesis, which extends from mid-embryogenesis to the second postnatal week, and adult hippocampal neurogenesis, which starts around post-natal day 14 (P14) and continues throughout life.

However, hippocampal neurogenesis continues to change considerably after P14 in mice as the DG transitions from developmental to adult rates of neuronal production. Indeed, a stereotypic pattern has been observed in most mammals; the numbers of new neurons generated in the DG are high in juveniles but decline rapidly in young adults and are maintained at low levels throughout adulthood (Amrein, 2015; Snyder, 2019). This transition from high levels of hippocampal neurogenesis in juveniles to lower and sustained levels in adults differs from an aging process because it occurs early in life, suggesting that it is adaptive. Interestingly, whether the same transition occurs in humans to extend neurogenesis beyond childhood has been disputed recently, highlighting the lack of understanding of the processes that preserve neurogenesis between infancy and old age in model species such as mice (Moreno-Jiménez et al., 2019; Sorrells et al., 2018).

Hippocampal NSCs sit atop the lineage hierarchy of neurogenesis, and their numbers also decline over time. Changes in NSC behavior are therefore likely to be involved in the transition to lower and sustainable levels of neurogenesis during adulthood. However, the behavior of adult hippocampal NSCs is still not well understood, and current models diverge on key points (Lazutkin et al., 2019). In particular, Encinas et al. (2011) described a “disposable stem cell model” where NSCs that have left quiescence progress rapidly through a series of neurogenic divisions without returning to quiescence and eventually differentiate into astrocytes, leading to a decrease in the NSC pool. Bonaguidi et al. (2011) proposed a contrasting “long-term self-renewal” model where many NSCs return to quiescence after dividing so that the NSC pool declines only slightly with age. Finally, we have proposed a model supporting heterogeneity in stem cell behavior where degradation of the pro-activation factor ASCL1 in proliferating stem cells allowed a subset of these cells to return to quiescence (Urbán et al., 2016).

Here we show that these different models are valid but describe NSC behavior at different stages throughout adult life. At the onset of adult neurogenesis, all proliferating NSCs are lost rapidly through differentiation, whereas by 6 months of age, more than half of the dividing NSCs return to quiescence. We determine that cells that have returned to quiescence (resting NSCs) have molecular and cellular properties distinct from quiescent cells that have never proliferated (dormant NSCs) and that they play an increasingly important part in maintaining NSC proliferation over time. Finally, we find that these coordinated changes are caused by an increase in post-translational degradation of the ASCL1 protein. Our results demonstrate that progressive and coordinated changes in NSC properties enable transition from high levels of neurogenesis coupled with stem cell depletion in juveniles to lower but sustainable levels of neurogenesis associated with stem cell self-renewal throughout adult life.

## Results

### Rapid NSC depletion in juvenile mice and reduced depletion in adults

**Figure 1:**
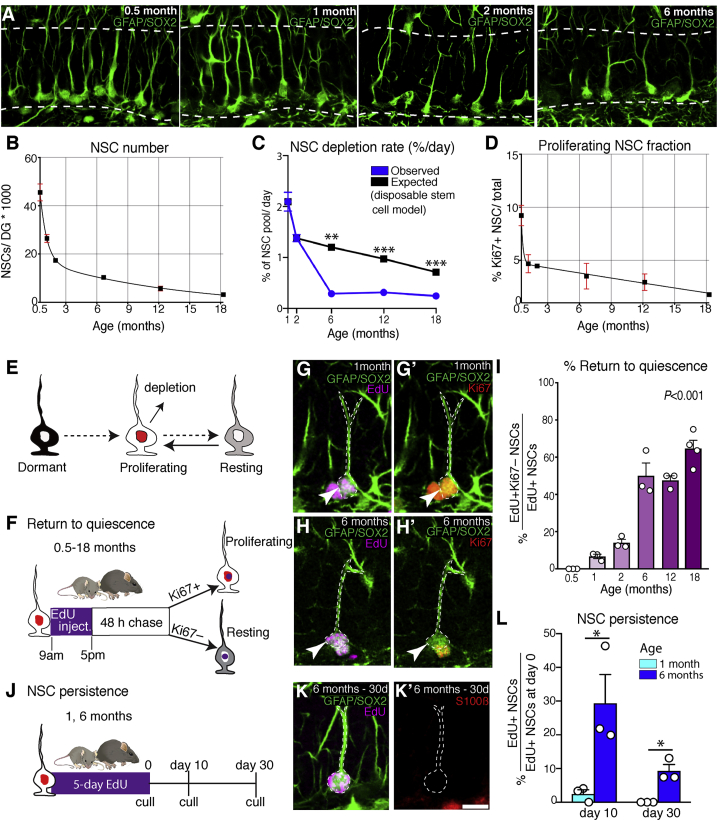
Proliferating NSCs increasingly return to quiescence with time (A) Images demonstrating that loss of hippocampal NSCs is rapid in young mice (0.5–2 months) but more gradual in adults (>2 months of age). (B) Quantification of NSC numbers from 0.5–18 months of age. (C) The rate of NSC depletion is lower than predicted by the disposable stem cell model after 2 months of age. (D) Quantification of the fraction of proliferating NSCs from 0.5–18 months of age. (E) Schematic of the different hippocampal NSC states. Dormant NSCs have never proliferated, whereas resting NSCs have returned to quiescence from a proliferating state. (F) Proliferating NSCs were labeled via EdU injections followed by a 48-h chase. (G) In young mice, EdU+ NSCs rarely returned to quiescence; most were Ki67+. (H) Return to quiescence increased in frequency with age (Ki67–). (I) Quantification of return to quiescence at different ages. (J) The ability of EdU-incorporating NSCs in 1- and 6-month-old mice to persist in the hippocampal niche was determined by measuring the fraction of EdU+ NSCs that remained as NSCs 10 and 30 days after a 5-day EdU labeling protocol. (K) Representative image of an EdU+ NSC that persisted for 30 days after labeling in a 6-month-old mouse. (L) Quantification of NSC persistence. Graphs show mean ± SEM. In (B)–(D), 3 mice were analyzed per time point, except at 12 months, where 8 mice were analyzed. Dots represent individual mice in (I) and (L). Statistics: t test in (C) and (L), and one-way ANOVA in (I). ^∗^p < 0.05, ^∗∗^p < 0.01, ^∗∗∗^p < 0.001. Scale bar (located in K'): 19 μm in (A), 10 μm in (G), (G'), (H), and (H'), and 11.25 μm in (K) and (K’).

We set out to examine the cellular properties of hippocampal NSCs from juvenile stages to old age. At the onset of adult neurogenesis in 0.5-month-old mice (Berg et al., 2019; Noguchi et al., 2019), there were approximately 46,000 NSCs per DG that underwent an immediate and rapid decline so that their number had more than halved in 2-month-old mice (Figures 1A and 1B). After this rapid loss of NSCs in juveniles, the depletion rate, defined as the percentage of the hippocampal NSC pool that was lost each day, slowed in young adults and reached much lower levels in 6-, 12-, and 18-month-old mice (Figure 1C). The disposable stem cell model proposed for hippocampal NSCs (Encinas et al., 2011) states that NSC activation leads to a series of rapid asymmetric divisions and eventual loss of the NSC through astrocytic or neuronal differentiation (Pilz et al., 2018). Because this model links NSC depletion to their activity, a lower rate of depletion might reflect a reduction in NSC activity. Indeed, the depletion rate decreased in proportion to the size of the proliferating (Ki67+) NSC pool in juveniles (between 1–2 months; Figures 1C and 1D). However, the depletion rate slowed considerably more than NSC activity in young adults (between 2 and 6 months of age) and became decoupled from proliferation so that it became substantially lower than predicted by the disposable stem cell model (Figure 1C). We therefore reasoned that proliferating NSCs may acquire distinct properties in early adulthood that function to slow depletion and preserve the NSC population throughout adult neurogenesis.

### Proliferating NSCs progressively acquire the capacity to return to quiescence

We investigated whether proliferating hippocampal NSCs might avoid depletion in young adults by returning to quiescence. We refer to proliferating NSCs that have returned to quiescence as resting NSCs to distinguish them from dormant NSCs that have remained quiescent since establishment of the DG niche (Figure 1E; Urbán et al., 2016). To label proliferating hippocampal NSCs, mice of different ages (0.5–18 months) were all given the thymidine analog 5-Ethynyl-2′-deoxyuridine (EdU) via injections and were culled after a 48-h chase (Figure 1F; Chehrehasa et al., 2009). EdU+ NSCs were identified as having remained cycling or having returned to a quiescent state depending on whether they co-expressed Ki67 protein. In 2-week-old mice, the hippocampal NSC niche is histologically mature and comprises a large population of mostly quiescent NSCs (~90%; Figure 1D; Berg et al., 2019; Noguchi et al., 2019). However, all EdU+ NSCs in 2-week-old mice remained cycling at the end of the chase period (EdU+Ki67+, 36 of 36), indicating that, at onset of adult neurogenesis, proliferating NSCs do not have the capacity to return to quiescence (Figures 1G and 1I). In 1-month-old mice, in contrast, we found that a small fraction (6.68% ± 1.17%) of EdU+ NSCs returned to quiescence (EdU+Ki67–). The fraction of proliferating NSCs that exited the cell cycle then increased in a gradual and substantial manner so that the majority of EdU+ NSCs returned to a quiescent state in mice by 6 months of age (Figures 1H and 1I).

We confirmed these results with independent approaches. First we labeled proliferating NSCs by genetic means, utilizing mice containing a *Ki67-creER*^*T2*^ allele (Basak et al., 2018) and a *tdTomato* cre-reporter (Madisen et al., 2010). In these Ki67^TD^ mice, transcription of creER^T2^ occurs in cells progressing through the cell cycle. We administered tamoxifen to 1- and 6-month-old Ki67^TD^ mice to label proliferating NSCs and culled the mice at the end of the injection period. Consistent with the EdU labeling experiment, proliferating NSCs (tdTomato+) returned to a quiescent state (Ki67–) at a greater rate in 6-month-old mice than in 1-month-old mice (Figures S1A–S1D).

Further, we confirmed the increased rate of return to quiescence with age by analyzing EdU-labeled NSCs with MCM2 labeling. *Mcm2* is highly transcribed throughout the cell cycle, including G_1_ phase (Braun and Breeden, 2007; Tsuruga et al., 1997; Young and Tye, 1997), unlike Ki67 (Miller et al., 2018). We determined that the MCM2 protein is extremely stable and that it is detected not only throughout the entire cell cycle but also for at least 72 h after a cell has exited the cell cycle (by returning to quiescence or differentiating), which is approximately twice as long as Ki67 (Figures S1E–S1H). Thus, although an EdU+ NSC that is Ki67– can have returned to G_0_ phase or be progressing through a G_1_ phase that is longer than 40 h, an EdU+ NSC that is MCM2– must have left the cell cycle and returned to quiescence for at least 72 h. We labeled proliferating cells in 1- and 6-month-old mice with EdU injections and quantified the number of EdU+ NSCs that were MCM2– 1 week later to allow sufficient time for MCM2 to degrade (Figure S1I). We found that, in 6-month-old mice, 20.9% of EdU+ NSCs retained their stem cell identity and were MCM2– and, thus, had returned to quiescence for at least 72 h (Figure S1J). This represented an ~200-fold increase over 1-month-old mice (Figure S1J). Moreover, the absolute number of EdU+MCM2− NSCs was 23-fold higher in 6-month-old mice than in 1-month-old mice (Figures S1K and S1L), despite the much lower levels of proliferation that occur with age. These independent lines of evidence demonstrate that proliferating hippocampal NSCs increasingly return to quiescence with time.

We reasoned that because proliferating NSCs increasingly return to quiescence rather than differentiating, they might persist as NSCs for longer in older mice, helping to preserve the NSC pool. To examine the long-term persistence of proliferating NSCs in the hippocampal niche, we labeled proliferating NSCs with EdU for 5 days in drinking water and examined their fate after 10 and 30 days (Figure 1J). In 1-month-old mice, only 2.46% ± 1.25% of EdU+ NSCs retained their NSC identity 10 days after labeling, and none retained it after 30 days (Figure 1L). Strikingly, in 6-month-old mice, there was an approximately 15-fold increase in the fraction of proliferating NSCs that persisted as NSCs after the 10-day chase (29.37% ± 8.5%), and a third of those (9.3% ± 1.89%) retained their NSC identity after the 30-day chase (Figures 1K and 1L). These findings demonstrate that proliferating hippocampal NSCs progressively acquire the capacity to return to quiescence, resulting in long-term self-renewal that supports maintenance of the NSC pool with age.

### Resting NSCs increasingly contribute to the proliferative NSC pool

**Figure 2:**
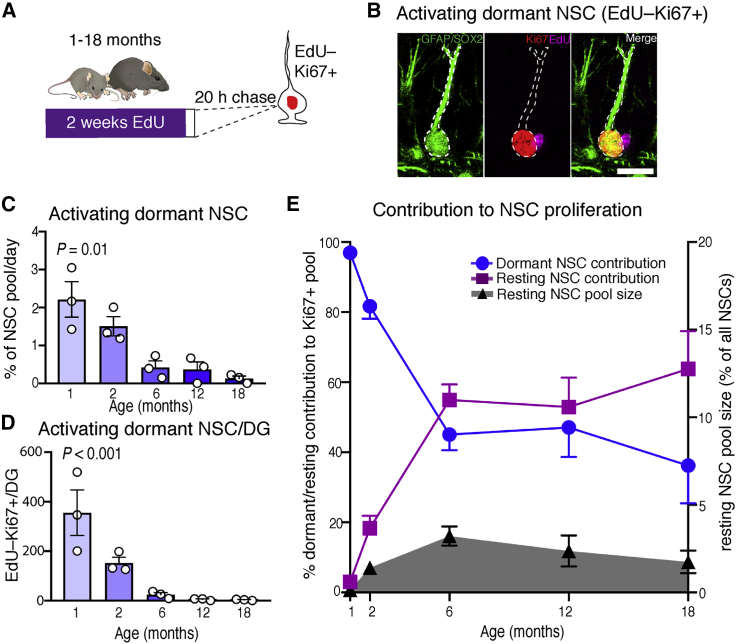
Resting NSCs increasingly contribute to the proliferative NSC pool with time (A) Mice received EdU via drinking water for 2 weeks to label proliferating and resting NSCs, followed by a 20-h chase. (B) Dormant NSCs that proliferated during the 20-h chase period were identified as EdU–Ki67+ NSCs. (C) The activation rate of dormant NSCs, normalized to the total size of the NSC pool, decreased with age, indicating deepening quiescence of this population. (D) The absolute numbers of EdU–Ki67+ cells also decreased with age. (E) The contribution of dormant NSCs to the proliferative NSC pool decreased with age, whereas the contribution of resting NSCs increased despite the resting NSC pool remaining relatively small even at advanced ages. Graphs show mean ± SEM. Dots represent individual mice in (C) and (D); in (E), 3 mice were analyzed per time point. Statistics: one-way ANOVA in (C) and (D). Scale bar in (B), 10 μm.

Next we wanted to find out whether dormant NSCs, which have not proliferated previously, may also alter their properties with time. To address this, we gave mice of different ages (1–18 months) EdU in the drinking water for 2 weeks and culled them after a 20-h chase. Resting NSCs do not remain quiescent over the long term, particularly in young mice (Figure 1L), and therefore most resting and proliferating NSCs are labeled by prolonged EdU exposure in these paradigms, meaning that the EdU– NSC population essentially corresponds to the dormant NSC pool. We found that the fraction of dormant NSCs that became active and proliferated during the 20-h chase period (i.e., EdU–Ki67+ NSCs; Figure 2B) declined progressively and substantially with age, indicating that dormant NSCs were progressing into a deeper state of quiescence over time (Figures 2C and 2D).

Even in 6- to 18-month-old mice, the numbers of resting NSCs remained small compared with the size of the dormant pool (Figure 2E). We therefore quantified the functional significance of resting NSCs by determining their contribution to the proliferative NSC pool at different ages. As expected, in juvenile mice where the resting pool is very small and the rate of activation of dormant cells is relatively high, the vast majority of proliferating NSCs arose from the dormant NSC pool. However, in 6- to 18-month-old mice, when the resting pool has grown and dormant cells have become more deeply quiescent, resting NSCs were the origin of 55%–63.8% of proliferating NSCs despite comprising only 1.5%–3% of the NSC population (Figure 2E). These findings imply that resting hippocampal NSCs are in a shallower state of quiescence and have a higher rate of activation than dormant NSCs. They also support a novel model where, with time, hippocampal NSC proliferation is generated increasingly from resting NSCs that have the capacity to shuttle between active and quiescent states, delaying NSC depletion and ensuring long-term maintenance of the NSC pool.

### Increasing numbers of NSC self-renewing divisions over time

**Figure 3:**
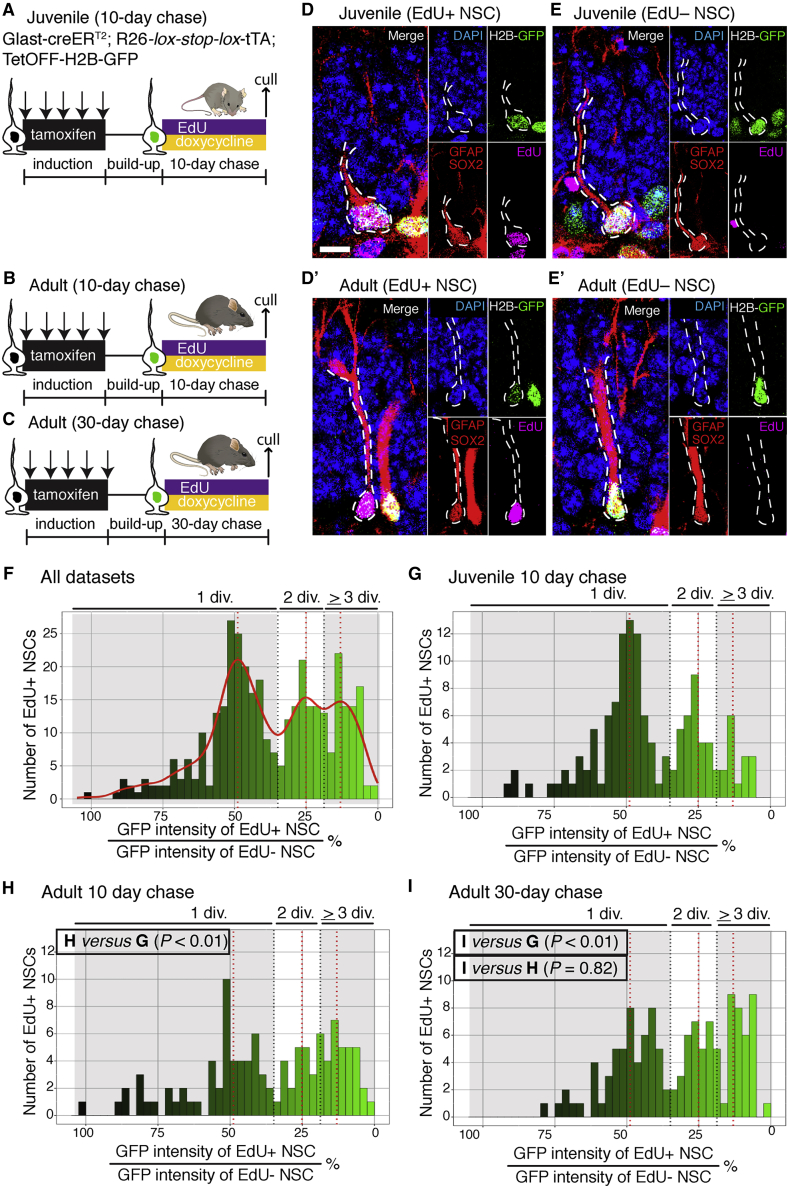
NSCs perform more self-renewing divisions with time (A) A juvenile cohort (P14) of H2B-GFP mice was injected with tamoxifen to induce GFP expression in NSCs. The mice received Dox for 10 days to stop label incorporation and EdU to mark dividing cells. (B) An adult cohort (5 months old) received the same treatment. (C) A second cohort of adult mice (4.5 months old) received Dox for 30 days to control for the increased time a proliferating NSC persists before depleting in adult mice. (D) The H2B-GFP label becomes diluted in EdU+ NSCs. (E) In quiescent EdU– NSCs, the label remained undiluted. (F) The GFP signal from EdU+ NSCs was categorized into discrete bins through automated analysis. These bins corresponded to the numbers of self-renewing divisions. (G) Label dilution profile of EdU+ NSCs from juvenile mice (10-day chase). (H) The label dilution profile of EdU+ NSCs from adult mice (10-day chase) showed increased self-renewal compared with juveniles. (I) Label dilution profile of EdU+ NSCs from adult mice (30-day chase) also showed increased self-renewal compared with juveniles. Statistics: Fisher’s exact test in (G)–(I). Scale bar (located in D): 15 μm in (D) and (E) and 10 μm in the subset panels in (D) and (E). div, divisions.

We next wondered whether returning to the resting state and escaping differentiation might provide NSCs with additional opportunities for self-renewing divisions. To address this possibility, we developed a mouse model to count the number of self-renewing divisions each hippocampal NSC undergoes prior to depleting from the niche. We used a labeling system based on the histone 2B (H2B)-green fluorescent protein (GFP) fusion protein, which accumulates in nuclei and is then repressed through doxycycline (Dox) administration, resulting in dilution of the GFP label following cell division (Kanda et al., 1998; Tumbar et al., 2004). We compared juvenile (1.5 month) and adult (6 month) cohorts in which the H2B-GFP label was targeted to NSCs using the *Glast* gene promoter (Figures 3A–3E; Mori et al., 2006).

We found that, at both ages, most NSCs underwent between 1 and 3 self-renewing divisions before losing their stem cell identity. However, the patterns of H2B-GFP dilution were different between ages, indicating different division patterns (Figures 3F–3I). For example, in a label dilution experiment where mice received Dox for identical lengths of time, 64% of NSCs self-renewed only once in juvenile mice (versus 50% in adults), whereas 12% of NSCs underwent three or more self-renewing divisions (versus 28% in adults) (Figures 3G and 3H). Thus, the increased self-renewal with time is an additional mechanism contributing to preservation of the NSC pool in adults.

### Mathematical modeling of time-dependent changes

To extend these observations, we built a mathematical model of hippocampal NSC population dynamics at different ages. For model fitting, we used the numbers of total NSCs (Figure 1B), proliferating NSCs (Ki67+, referred to as active cells throughout modeling section; Figure 1D), and resting NSCs (EdU+Ki67–; Figure 2E) from mice 0.5–12 months of age. In our model (Figure S2A; Data S1), we found that a decreasing activation rate provided a good fit for the numbers of total NSCs and active NSCs, as already found in our previous model of hippocampal NSC dynamics (Ziebell et al., 2018). However, the model was a poor fit for resting NSCs (the quiescent label-retaining cell population) because it did not take into account that dormant and resting cells could have different activation rates (Figure S2B). We therefore retained the time-dependent activation rate from the initial model but allowed the absolute value of the activation rate to differ between resting NSCs and dormant NSCs. This change resulted in dramatic improvement of the fit of the model (Akaike information criterion [AIC] = 53 compared with 120 for the model where both populations have the same activation rate; Figures S2B and S2C). In the top-ranked model (AIC = 36; Figure 2D), resting NSCs across the first 6 months of life had a median activation rate 29.2-fold higher than that of dormant NSCs. We tested a variety of other scenarios (e.g., time-dependent cell cycle duration), and in isolation these models were poor (Table S1; Data S1). Likewise, when we added time-dependent self-renewal and time-dependent cell cycle duration to the model of best fit, they did not make improvements (AIC > 36), suggesting that these features contribute less to time-dependent changes than activation rates. Furthermore, refitting of the model to the data under the assumption that activation rates, proliferation rate, and self-renewal are age dependent predicts that age-related changes of proliferation rate and self-renewal are small compared with the changes in activation rates (Data S1). Accumulation of resting NSCs with a high activation rate and the decreasing activation rate of dormant NSCs are the main features that explain the overall increased contribution of resting NSCs to proliferation (Table S1) and the decreased depletion of NSCs over time.

### Single-cell RNA sequencing and pseudotemporal ordering demonstrate state- and age-dependent changes to hippocampal NSC quiescence

**Figure 4:**
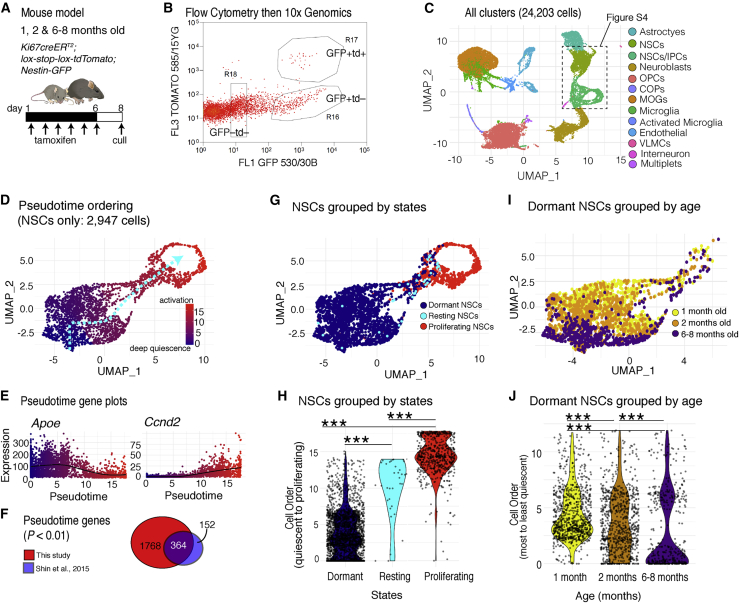
The quiescence depth of hippocampal NSCs is dependent on the proliferation history of the stem cell and the age of the mouse (A) Cohorts of 1-, 2-, and 6- to 8-month-old Ki67^TD-NES^ mice were given tamoxifen and culled after 8 days, and then the DG was dissociated. (B) GFP+tdTomato+ and GFP+tdTomato− cells were collected by flow cytometry and sequenced using a 10x Genomics platform. (C) Uniform Manifold Approximation and Projection (UMAP) plot showing the 24,203 cells sequenced from 8 experiments. (D) After two iterations of subsetting and re-clustering, a dataset of 2,947 NSCs was ordered using Slingshot, revealing a pseudotime trajectory from the most quiescent NSCs (blue) to proliferating NSCs (red). (E) Pseudotime progression is correlated negatively with *Apoe* expression and positively with *Ccnd2* expression. (F) Strong concordance between genes associated with this pseudotime trajectory and those from Shin et al. (2015). (G) UMAP plot showing the locations of dormant (quiescent and GFP+tdTomato–), resting (quiescent and GFP+tdTomato+), and proliferating NSCs (cell cycle gene expression and GFP+tdTomato+/–). (H) Plotting pseudotime positions reveals that resting NSCs are in a shallower state of quiescence than dormant NSCs. (I) UMAP plot showing the location of dormant cells according to mouse age. (J) Plotting pseudotime positions of dormant NSCs grouped by age reveals a progressive increase in quiescence depth. Dots in UMAP and violin plots represent individual cells. Statistics: Mann-Whitney *U* test in (H) and (J). ^∗∗∗^p < 0.001.

The age-dependent changes in hippocampal NSC properties and in particular the different activation rates of resting and dormant NSCs warranted transcriptomic characterization to identify the molecular mechanisms underlying these properties. To achieve this, we crossed the Nestin-GFP mouse line that labels NSCs (Mignone et al., 2004) with Ki67^TD^ mice to label proliferating cells and their progeny (Basak et al., 2018). The generated mice (hereafter called Ki67^TD-NES^) were injected with tamoxifen at 1-month, 2-months, and 6–8 months of age to irreversibly label the progeny of proliferating cells with red fluorescence, their DG was disassociated, and GFP+tdTomato– and GFP+tdTomato+ cells were sorted by flow cytometry and sequenced with the 10x Genomics platform (Figures 4A and 4B; Figure S3). The dataset of 24,203 cells was subsetted into NSCs and intermediate progenitor cells (IPCs) (Figure 4C; Figures S4A and S4B) and then re-clustered to exclude IPCs and isolate only quiescent and proliferating NSCs (Figures S4C and S4D). From this final dataset of 2,947 NSCs, we used the trajectory tool Slingshot to infer progression along a pseudotime curve from quiescence to activation (Figures 4D and 4E; Street et al., 2018), which we validated against an existing pseudotime analysis of 123 hippocampal progenitors (Shin et al., 2015; Figure 4F).

We then analyzed the positions along the pseudotime axis of NSCs in different states (proliferating, resting, and dormant) and of different ages. We identified proliferating NSCs in G_2_/S/M phase based on cell cycle scoring algorithms (Butler et al., 2018). However, to distinguish proliferating NSCs in G_1_ phase from quiescent NSCs in G_0_ phase among recombined (tdTomato+) cells, we used the expression of *Mcm* and other G_1_/S-phase genes (Figure S5; STAR methods). Supporting our thresholding approach, resting NSCs expressed an independent set of G_1_/S-phase genes at similarly low levels as dormant cells (Figure S5D) and had total mRNA levels that were half of those of proliferating NSCs and equivalent to those of dormant NSCs, indicative of a quiescent state (Figure S5E). As predicted from the 2-week EdU labeling experiments (Figure 2E), resting NSCs were rare (Figure 4G). They segregated along the pseudotime axis at positions intermediate between proliferating NSCs and dormant NSCs (tdTomato– and quiescent), with only a minority of resting cells occupying deeply quiescent positions (Figures 4G and 4H). Our single-cell transcriptomics analysis therefore shows that proliferating NSCs that return to a state of quiescence are among the shallowest quiescent NSCs.

The effect of age on the depth of dormant NSC quiescence was also striking (Figures 4I and 4J). The distribution of NSC pseudotime positions from 1-month-old mice was clearly overrepresented in shallower states of quiescence, whereas NSCs at 2 months of age were distributed evenly across the quiescence spectrum, and NSCs from 6- to 8-month-old mice were over-represented in deeper states of quiescence. Thus, dormant hippocampal NSCs demonstrate an early and continuing progression into deeper quiescence. The single-cell RNA sequencing (scRNA-seq) data support the EdU labeling data and mathematical modeling reporting the existence of a small population of resting NSCs that exist in a shallow quiescent state and the deepening quiescence of dormant NSCs.

### Division history affects the molecular properties of quiescent hippocampal NSCs

**Figure 5:**
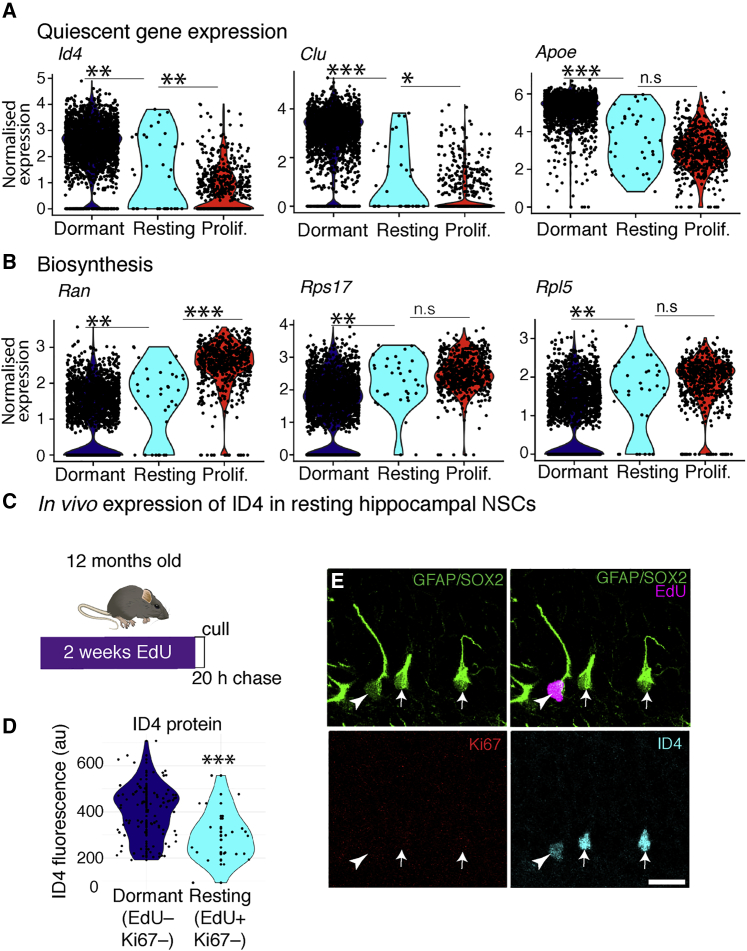
Resting NSCs display a distinct transcriptional profile indicative of a shallow quiescent state (A) Resting NSCs exhibit lower expression of quiescence marker genes than dormant NSCs (scRNA-seq). (B) Resting NSCs have increased expression of genes involved in biosynthesis compared with dormant NSCs (scRNA-seq). (C) To validate the differential gene expression analysis, 12-month-old mice received EdU for 2 weeks and were culled after a 20-h chase. (D) Resting NSCs had reduced staining intensity for ID4 than dormant NSCs. (E) Image of resting NSC (EdU+Ki67–, arrowheads) and dormant NSCs (EdU–Ki67–, arrows) with ID4 co-staining. Graphs are violin plots. Dots in violin plots represent individual cells. Statistics: t test performed on Pearson’s residuals with false discovery rate (FDR)-corrected p value in (A) and (B) and t test in (D). ^∗^p < 0.05, ^∗∗^p < 0.01, ^∗∗∗^p < 0.001. Scale bar in (E), 15 μm. a.u., arbitrary units.

The previous findings suggest that resting NSCs might diverge molecularly from dormant NSCs. To directly address this, we used the scRNA-seq data to compare gene expression in the different NSC populations. Gene Ontology analysis of differential gene expression between resting and dormant NSCs (880 genes; Table S3), showed that resting NSCs had reduced expression of genes associated with lipid metabolism, suggesting shallower quiescence because NSCs require *de novo* lipogenesis and fatty acid oxidation to maintain quiescence (Knobloch et al., 2013, 2014; Figure 5A). Resting NSCs also showed increased expression of genes associated with ribosomal biogenesis, mRNA processing, and translation elongation, demonstrating upregulation of protein biosynthesis pathways, which has been associated with exit from quiescence (Figure 5B; Cabezas-Wallscheid et al., 2017). Furthermore, multiple genes/pathways that have been associated with maintenance of quiescence, such as *Clu* (Basak et al., 2018), *Hopx* (Berg et al., 2019), *Notch2* (Engler et al., 2018), and *Id4* (p < 0.01) (Blomfield et al., 2019), were downregulated in resting NSCs compared with dormant NSCs (Figure 5A; Table S3). We validated the dataset by confirming that expression of ID4 was downregulated at a protein level in resting versus dormant NSCs (Figures 5C–5E). We extended this analysis to genes expressed differentially between resting and proliferating NSCs. Excluding cell cycle genes that were upregulated uniquely in proliferating NSCs, many of the aforementioned quiescence-specific genes were expressed in resting NSCs at an intermediate level between dormant and proliferating states (Figures 5A and 5B; Table S4). Thus, although resting NSCs are quiescent, they show increased expression of metabolic and biosynthetic pathways and are primed toward re-activation.

### Declining ASCL1 levels explain time-dependent changes in quiescence

The fact that the properties of dormant, resting and proliferating NSCs change coordinately over time suggested that a common molecular mechanism might underlie these changes. The transcription factor ASCL1 was a strong candidate to contribute to the changing properties of NSCs; induction of ASCL1 expression is required for activation of quiescent NSCs (Andersen et al., 2014), and a reduction of ASCL1 protein levels allows proliferating hippocampal NSCs to return to a resting state (Urbán et al., 2016) and increases the likelihood that embryonic NSCs proliferate rather than differentiate (Imayoshi et al., 2013). We reasoned that the changing properties of hippocampal NSCs might reflect progressively lower expression levels of ASCL1 over time.

**Figure 6:**
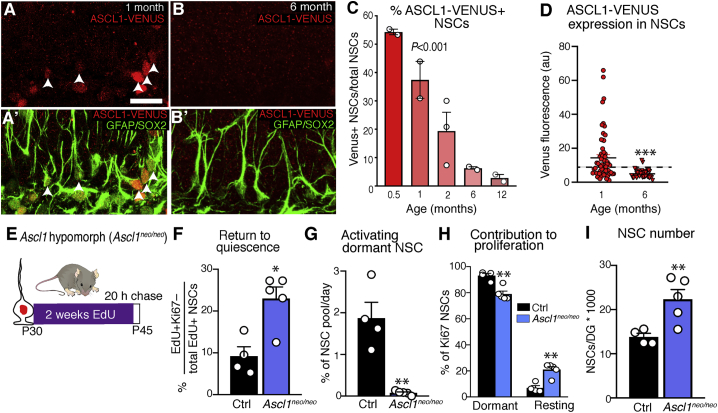
Ascl1 protein levels decrease with time and cause progressive changes in NSC behavior (A) ASCL1-VENUS staining in hippocampal NSCs from 1-month-old mice (arrowheads indicate positive cells) using an anti-GFP antibody. (B) ASCL1-VENUS staining in NSCs from 6-month-old mice. (C) Fewer NSCs are positive for ASCL1-VENUS with age. (D) The expression intensity of the ASCL1-VENUS protein also decreases with age. (E) *Ascl1*^neo/neo^ and control mice were given EdU to label proliferating and resting NSCs and were culled following a 20-h chase. (F) Hippocampal NSCs were returning more to quiescence after proliferating (EdU+Ki67−) in *Ascl1*^neo/neo^ mice than in controls. (G) Dormant NSCs activated less frequently in *Ascl1*^neo/neo^ mice. (H) Resting NSCs contributed more to the proliferative NSC pool in *Ascl1*^neo/neo^ mice than in controls. (I) *Ascl1*^neo/neo^ mice had more NSCs than controls. Graphs represent the mean ± SEM. Dots represent individual mice, except in (D), where dots represent individual cells (minimum of 20 cells analyzed per mouse, 2 mice per age). Statistics: one-way ANOVA in (C) and t test in (D) and (F)–(I). ^∗^p < 0.05, ^∗∗^p < 0.01, ^∗∗∗^p < 0.001. Scale bar (located in A): 21.9 μm in (A) and (B). a.u., arbitrary units.

*Ascl1* is transcribed by most NSCs (Blomfield et al., 2019). However, because of low protein levels as a result of degradation induced by the E3 ubiquitin ligase HUWE1 (Urbán et al., 2016) and by Inhibitor of differentiation/DNA binding (ID) factors (Blomfield et al., 2019), ASCL1 protein cannot be detected by immunolabeling in quiescent NSCs and can only be seen when ASCL1 protein levels are at their highest; i.e., in proliferating NSCs. To increase ASCL1 detection, we examined its expression in a mouse line that expresses an ASCL1-VENUS fusion protein (Imayoshi et al., 2013). Labeling of the fusion protein with anti-GFP antibodies greatly enhanced detection of ASCL1 protein so that low levels were readily detectable even in quiescent NSCs (Figure S6). We found that there was a progressive but dramatic reduction in the proportion of NSCs (including quiescent NSCs) that express detectable levels of ASCL1-VENUS throughout adulthood. 54% of NSCs expressed ASCL1-VENUS in 0.5-month-old mice, 19% in 2-month-old mice, and 3% in 12-month-old mice (Figures 6A–6C). There was a parallel reduction in the intensity of ASCL1-VENUS fluorescence in NSCs from 6-month-old mice relative to 1-month-old mice (Figure 6D). Thus, the observed changes in hippocampal NSC properties with age correlate with a reduction in ASCL1 protein.

To establish causality, we utilized an *Ascl1* hypomorphic mouse line (*Ascl1*^neo/neo^) that expresses reduced levels of *Ascl1* in adult hippocampal NSCs (Andersen et al., 2014). We labeled proliferating, resting, and dormant NSCs in 1-month-old *Ascl1*^neo/neo^ mice by EdU retention and Ki67 labeling as before (Figure 6E). Compared with age-matched controls, EdU+ NSCs in hypomorphic mice were 2.5 times more likely to return to quiescence (Figure 6F), and dormant NSCs were 24 times less likely to exit quiescence (Figure 6G). As a result, resting NSCs contributed more to the proliferative pool in *Ascl1*^neo/neo^ mice than in controls (Figure 6H) and the total number of NSCs was increased, presumably because of a lower depletion rate (Figure 6I). Therefore, hippocampal NSCs in *Ascl1*^neo/neo^ mice display the behavior of NSCs from older wild-type mice, which provides direct evidence that the declining levels of ASCL1 drive the time-dependent changes in NSC behavior and the resulting reduction in NSC depletion rate.

### Declining ASCL1 levels are due to increased post-translational degradation

**Figure 7:**
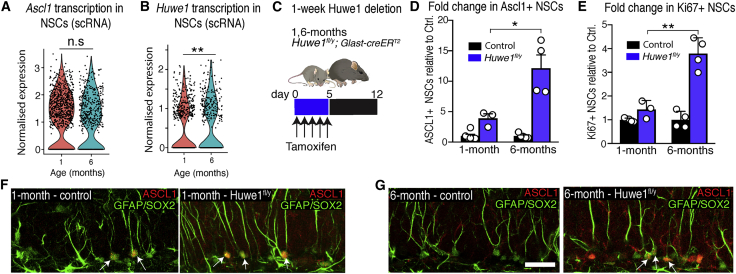
Expression and activity of *Huwe1* increases with time (A) *Ascl1* transcript levels in NSCs are largely unchanged between young and adult mice (scRNA-seq). (B) The expression of the ubiquitin-ligase *Huwe1* increases in older mice (scRNA-seq). (C) 1- and 6-month-old *Huwe1*^*fl/y*^ and control mice were culled 1 week after receiving tamoxifen. (D) The fold change increase in ASCL1+ NSCs in *Huwe1*^*fl/y*^ mice (relative to controls) was larger in the older cohort. (E) The fold change increase in Ki67+ NSCs in *Huwe1*^*fl/y*^ mice (relative to controls) was also larger in the older cohort. (F) Images of control and *Huwe1*^*fl/y*^ mice at 1 month of age; arrows indicate ASCL1+ NSCs. (G) Images of control and *Huwe1*^*fl/y*^ mice at 6 months of age; arrows indicate ASCL1+ NSCs. Graphs represent mean ± SEM or are violin plots in (A) and (B). Dots represent single cells in (A) and (B) and individual mice in (D) and (E). Statistics: t test performed on Pearson’s residuals with FDR-corrected p value in (A) and (B) and t test in (D) and (E). ^∗^p < 0.05, ^∗∗^p < 0.01. Scale bar (located in G): 28 μm in (F) and (G).

In our scRNA-seq analysis of hippocampal NSCs in 1-month-old and 6-month-old mice, the levels of *Ascl1* transcripts did not change significantly (Figure 7A). We therefore reasoned that the steep time-dependent decline in ASCL1 protein levels must be primarily due to altered post-translational regulation, such as through increased expression or activity of ID proteins or HUWE1, which promote targeting of ASCL1 for proteasomal destruction (Blomfield et al., 2019; Urbán et al., 2016). Indeed, expression of *Huwe1* increased in 6-month-old NSCs relative to its expression in 1-month-old NSCs (Figure 7B), whereas, among *Id* genes, *Id4* was upregulated but *Id1–Id3* were downregulated with age (Table S5). We therefore focused on the role of HUWE1 in driving the time-dependent change in ASCL1 protein expression. HUWE1-dependent degradation of ASCL1 has been shown to allow hippocampal NSCs to stop dividing and return to a resting state (Urbán et al., 2016), and it also suppresses activation of dormant NSCs (Figures S7A and S7B). Thus, increased expression of *Huwe1* and/or an increase in efficiency of HUWE1-mediated ASCL1 degradation with age might contribute to the progressive reduction in ASCL1 levels.

To test whether HUWE1 activity increases with age, we deleted *Huwe1* from hippocampal NSCs by administering tamoxifen to *Huwe1*^fl/y^; *Glast*-*creER*^*T2*^ mice at 1- and 6-months of age and analyzed the mice 1 week later (Figure 7C). The expected increase in the number of NSCs expressing ASCL1 was substantially larger in 6-month-old mice (12.1-fold increase) than in 1-month-old mice (3.9-fold increase) when normalized to age-matched controls (Figure 7D; Figure S7D). Similarly, the number of Ki67+ NSCs increased by a larger amount in 6-month-old mice (3.8-fold) than in 1-month-old mice (1.4-fold) (Figure 7E; Figure S7E), indicating that HUWE1 activity, which eliminates ASCL1 expression and suppresses NSC proliferation, is greater in adult than in juvenile mice. Finally, we asked whether *Huwe1* has a role in long-term maintenance of the NSC pool and whether this, too, is age dependent (Figure S7F). We found that the decline in NSC number was small 2 months after *Huwe1* deletion from 1-month-old mice but significantly larger 2 months after deletion at 5 months of age (Figures S7G and S7H). These results demonstrate that the expression and activity of *Huwe1* increase with time to reduce ASCL1 protein levels and mediate the associated changes in quiescence that serve to preserve the NSC pool during adult neurogenesis.

## Discussion

The changes in NSC behaviors we identify serve to preserve the NSC pool and long-term adult neurogenesis and are therefore distinct from stem cell exhaustion, which occurs during aging. Most of the changes we describe occur in the first 6 months of life when mice are of breeding age, suggesting an adaptive function. Aging is defined by functional decline and accumulation of cellular damage, resulting in loss of fitness (López-Otín et al., 2013). In contrast, we found that the reduction of NSC numbers during this period is not associated with loss of NSC function. On the contrary, the number of self-renewing divisions increases. Moreover, our transcriptional analysis of NSCs in 1-month-old and 6-month-old mice shows little change over time in inflammatory, mitochondrial, and lysosomal pathways that typify stem cell aging (Leeman et al., 2018). Instead, we propose that hippocampal NSCs progress, during juvenile and early adult stages, through a transition between developmental neurogenesis, characterized by large NSC numbers and high rates of neuronal production, and proper adult neurogenesis, characterized by smaller numbers of NSCs and lower rates of neuronal production.

Adult tissue stem cell compartments often have several stem cell populations that differ in their level of quiescence (Barker et al., 2010). Previous experiments have demonstrated that NSCs in the adult niches of the V-SVZ and DG lie on a trajectory from quiescence to activation, including a population of primed NSCs in the V-SVZ (Llorens-Bobadilla et al., 2015; Shin et al., 2015). However, whether all NSCs operate along the same trajectory, whether the trajectory can be reversed, and whether subpopulations of NSCs along this trajectory can be linked to specific cellular behaviors (i.e., returning to quiescence) has remained unclear. We find that hippocampal NSCs that have recently divided and returned to a resting state are in a much shallower quiescent state than NSCs that have never left quiescence. Although resting NSCs present lower expression levels of quiescence-associated transcription factors and higher levels of metabolic genes than dormant NSCs, they nevertheless are able to remain quiescent because of active degradation of the pro-activation factor ASCL1, which prevents NSC activation and cell cycle re-entry (Urbán et al., 2016). However, the reduced expression of quiescence-associated genes maintains resting NSCs in shallow quiescence, which allows them to reactivate much more readily than dormant cells and sustain NSC proliferation and neurogenesis over the long term.

Our data suggest that current conflicting models of hippocampal NSC behavior can be reconciled by considering changes in behavior over time. We find that NSCs uniformly adhere to the disposable stem cell model in juvenile mice (Encinas et al., 2011). Thereafter, heterogeneity emerges (Urbán et al., 2016), so that some hippocampal NSCs acquire the capacity to return to quiescence and long-term self-renewal increases, as reported by Bonaguidi et al. (2011). Importantly, the age of the mice used in the original studies might have contributed to their divergent conclusions. Two-month-old mice were used in the bromodeoxyuridine (BrdU) labeling experiments that originated the disposable stem cell model (Encinas et al., 2011), and 10-week-old mice were used when live imaging led to similar conclusions (Pilz et al., 2018). Three- to four-month-old mice were used in labeling experiments where a subpopulation of hippocampal NSCs were found to return to quiescence (Urbán et al., 2016), whereas a substantial part of the clonal experiments that posited long-term self-renewal was analyzed in 1-year-old mice (Bonaguidi et al., 2011). These observations suggest that use of mice of different ages contributed to development of contradictory models of hippocampal NSC behavior. The effect of time we describe here renders these models compatible.

We attribute the changes in hippocampal NSC dynamics throughout adulthood to the declining levels of ASCL1 protein. We found that the reduction in ASCL1 levels with age was due to increased post-translational degradation by HUWE1. The mechanisms leading to transcriptional upregulation of *Huwe1* and/or its increased activity are unclear. It has been shown that the phosphorylation status of substrates can affect HUWE1-mediated ubiquitination (Forget et al., 2014). Interestingly, ASCL1 stability is phospho dependent (Ali et al., 2014), and we identified a number of phosphatases and kinases that are expressed differentially in NSCs between adults and juveniles (Table S5). Identifying niche signals that control the phosphorylation status of ASCL1 and its interaction with HUWE1 might provide insights into timing mechanisms that drive changes in hippocampal NSC behavior during transition from developmental to adult neurogenesis. Our results demonstrate how a series of early, progressive, and coordinated changes in hippocampal NSC properties function to preserve proliferation beyond the juvenile period in mice. Whether similar mechanisms are present in humans to extend neurogenesis beyond childhood warrants investigation.

### Limitations of study

There are several limitations in the present study. First, our analysis of the emergence of resting NSCs and the deepening quiescence of dormant NSCs with age was restricted to scRNA-seq and nucleotide retention analysis. Further investigations should determine whether these age-dependent states involve lasting reorganization of chromatin, especially considering that ASCL1 is a pioneer transcription factor (Raposo et al., 2015). Second, the resolution of the H2B-GFP dilution method could not distinguish between 3 and more self-renewing divisions. Therefore, in future studies, the magnitude of the age-dependent increase in self-renewal would be best quantified by intravital live-imaging approaches.

## STAR★Methods

### Key resources table

**Table:** 

REAGENT or RESOURCE	SOURCE	IDENTIFIER
**Antibodies**		

Mouse monoclonal anti-Ascl1	BD PharMingen	RRID:AB_396479
Rat monoclonal anti-GFAP	Invitrogen	RRID:AB_86543
Chicken polyclonal anti-GFP	Abcam	RRID:AB_300798
Mouse monoclonal anti-Ki67	BD Biosciences	RRID:AB_393778
Rat monoclonal anti-Sox2	Invitrogen	RRID:AB_11219471
Goat polyclonal anti-tdTomato	Sicgen	RRID:AB_2722750
Rabbit polyclonal anti-MCM2	Cell Signaling	RRID:AB_2142134
Rabbit polyclonal anti-ID4	BioCheck	RRID:AB_2814978
Rabbit monoclonal anti S100b-647	Abcam	RRID:AB_2868562
Goat polyclonal anti-DCX	Santa Cruz Biotechnology	RRID:AB_2088494
Alexa Fluor 488-affiniPure F(ab’)2 Donkey anti-mouse IgG	Jackson ImmunoResearch	RRID:AB_2340849
Alexa Fluor 488-affiniPure F(ab’)2 Donkey anti-rabbit IgG	Jackson ImmunoResearch	RRID:AB_2340619
Alexa Fluor 488-affiniPure F(ab’)2 Donkey anti-rat IgG	Jackson ImmunoResearch	RRID:AB_2340686
Alexa Fluor 488-affiniPure Donkey anti-ch IgY	Jackson ImmunoResearch	RRID:AB_2340375
Alexa Fluor Cy3-affiniPure F(ab’)2 Donkey anti-mouse IgG	Jackson ImmunoResearch	RRID:AB_2340817
Alexa Fluor Cy3-affiniPure F(ab’)2 Donkey anti-rat IgG	Jackson ImmunoResearch	RRID:AB_2340669
Alexa Fluor Cy3-affiniPure F(ab’)2 Donkey anti-rabbit IgG	Jackson ImmunoResearch	RRID:AB_2313568
Alexa Fluor Cy3-affiniPure F(ab’)2 Donkey anti-goat IgG	Jackson ImmunoResearch	RRID:AB_2340413
Donkey anti-Rat IgG Alexa Fluor 594	ThermoFisher Scientific	RRID:AB_2535795
Alexa Fluor 647-affiniPure F(ab’)2 Donkey anti-mouse IgG	Jackson ImmunoResearch	RRID:AB_2340866
Alexa Fluor 647-affiniPure F(ab’)2 Donkey anti-rabbit IgG	Jackson ImmunoResearch	RRID:AB_2340625
Alexa Fluor 647-affiniPure F(ab’)2 Donkey anti-rat IgG	Jackson ImmunoResearch	RRID:AB_2340695
Alexa Fluor 647-affiniPure F(ab’)2 Donkey anti-goat IgG	Jackson ImmunoResearch	RRID:AB_2340438

**Chemicals, peptides, and recombinant proteins**		

Doxycycline	Sigma-Aldrich	Cat# D9891
Tamoxifen	Sigma-Aldrich	Cat# T5648
Cornflower oil	Sigma-Aldrich	Cat# C8267
5-Ethynyl-2′-deoxyuridine	Santa Cruz Biotechnology	Cat# sc-284628
Recombinant Mouse BMP-4	R&D Systems	Cat# 5020-BP-010
Recombinant Murine FGF2	Peprotech	Cat# 450-33
Laminin	Sigma-Aldrich	Cat# L2020
Heparin	Sigma-Aldrich	Cat# H3393-50KU
DMEM/F12 + GLUTAMAX	ThermoFisher Scientific	Cat# 31331093
DMEM/F12 without phenol red	ThermoFisher Scientific	Cat# 21041025
DAPI	ThermoFisher Scientific	Cat# D1306

**Critical commercial assays**		

Click-iT® EdU Alexa Fluor 647 Imaging Kit	Invitrogen	Cat# C10340
Click-iT® PLUS EdU Alexa Fluor 647 Imaging Kit	Invitrogen	Cat# C10640
Neural Tissue dissociation kit (P)	Milteny Biotec	Cat# 130-092-628

**Deposited data**		

scRNA-sequencing data	This study	GEO: GSE159768
Code to analyze scRNA-sequencing data	This study	https://github.com/harrislachlan/lifelong_stemcells
scRNA-sequencing data	Shin et al., 2015	GEO: GSE71485

**Experimental models: cell lines**		

Primary adult hippocampal wildtype neural stem cell line no. 5	Francois Guillemot	Blomfield et al., 2019

**Experimental models: organisms/strains**		

Slc1a3^tm1(cre/ERT2)Mgoe^ (Glast-CreERT2)	Mori et al., 2006	MGI:5466676
Gt(ROSA)26Sor^tm1(EYFP)Cos^ (RYFP)	Srinivas et al., 2001	MGI:2449038
Gt(ROSA)26Sor^tm9(CAG-tdTomato)Hze^ (tdTomato)	Madisen et al., 2010	MGI: 3809523
Ascl1^tg1(venus)Rik^ (Ascl1Venus)	Imayoshi et al., 2013	MGI: 6369044
Huwe1^tm1Alas^ (Huwe1^fl^)	Zhao et al., 2008	MGI: 4439480
C.129P2(B6)-*Gt(ROSA)26Sor*^*tm1(tTA)Roos*^/J (*R26tTa*)	Wang et al., 2008	Cat# (JAX Stock): 008603
Tg(Nes-EGFP)33Enik (Nestin::GFP)	Mignone et al., 2004	MGI: 5523870
Mki67^tm2.1(cre/ERT2)Cle^ (Ki67-creER^T2^)	Basak et al., 2018	MGI: 5816737
Tg(tetO-HIST1H2BJ/GFP)47Efu (H2B::GFP)	Kanda et al., 1998	MGI: 3044190

**Software and algorithms**		

Seurat	Stuart et al., 2019	RRID:SCR_007322 (v3.1.2)
Slingshot	Street et al., 2018	RRID:SCR_017012 (v1.2.0)
Multimode	Ameijeiras-Alonso et al., 2018	https://cran.r-project.org/web/packages/multimode/ v1.4
GraphPad Prism 8	GraphPad Software	RRID:SCR_002798
FIJI v1.0	Schindelin et al., 2012	RRID:SCR_003070

### Resource availability

#### Lead contact

Further information and requests for reagents should be directed to and will be fulfilled by the Lead Contact, François Guillemot (francois.guillemot@crick.ac.uk).

#### Materials availability

This study did not generate new reagents.

#### Data and code availability

The accession number for the raw RNA sequencing data reported in this paper is GEO: GSE159768. The code to reproduce the analyses can be found at Github (https://github.com/harrislachlan/lifelong_stemcells).

### Experimental model and subject details

Wild-type animals in this study (Figures 1 and 2) were on a C57BL/6J genetic background (000664, The Jackson Laboratory). In the scRNA-seq data (Figures 4 and 5) and in a limited number of genetic and EdU-labeling experiments (Figure 1I; Figures S1A–S1D) mice were on a C57BL/6J/ CD1 mixed background heterozygous for the following transgenes, *Ki67*-*creER*^*T2*^ (Basak et al., 2018), *Nestin-GFP* (Mignone et al., 2004) and *tdTomato* (Madisen et al., 2010). In contrast, all other transgenic mice were maintained on a mixed genetic background with littermates serving as controls. Mice with conditional *Huwe1* (Zhao et al., 2008), *Ascl1*^neo/neo^ (Andersen et al., 2014), *R26tTa* (Wang et al., 2008), and *YFP* (Srinivas et al., 2001) alleles have been described before; as have the cre-driver lines *Glast*-creER^T2^ (Mori et al., 2006), and the reporter lines *Ascl1*-*venus* (Imayoshi et al., 2013), and *H2B*-*GFP* (Kanda et al., 1998). Both male and female mice were used throughout the study, an exception to this was for the x-linked *Huwe1* conditional allele, where only males were used. The effects seen throughout the study were consistent across sexes.

All experimental protocols involving mice were performed in accordance with guidelines of the Francis Crick Institute, national guidelines and laws. This study was approved by the UK Home Office (PPL PB04755CC). Throughout the study, mice were housed in standard cages with a 12 h light/dark cycle and *ad libitum* access to food and water.

### Method details

#### Mathematical modeling

Details of mathematical modeling are found in Data S1.

#### Cell culture

Adherent cultures of primary hippocampal neural stem and progenitors cells (AHNSC line #5) were propagated in basal media containing 20ng/ml FGF (Peprotech) (Blomfield et al., 2019). Once cells had reached 70% confluence, quiescence was induced with the addition of BMP4 (R&D) at 20ng/ml, and media was replaced every 3 days.

#### Preparation and sectioning of mouse brain tissue

Mice were perfused transcardially with phosphate buffered saline (PBS), followed by 4% paraformaldehyde (10-20 mL) and post-fixed for 16-24 h before long term storage in PBS with 0.02% sodium azide, at 4°C. Brains were sectioned in a coronal plane at 40 μm using a vibratome (Leica). The entire rostral-caudal extent of the hippocampus was collected in a 1 in 12 series.

#### Antibodies, immunofluorescence, cell counts

At least 1 series per mouse (5-6 sections), per experiment, was stained and analyzed. For experiments requiring the detection of fixation-sensitive antigens (ASCL1, TBR2, Ki67, MCM2) free-floating sections were subjected to heat-mediated antigen retrieval in sodium citrate solution (10 mM, pH 6.0) at 95°C for 10 min. A shorter duration of heat-retrieval was used (2 min) if the detection of GFP was required, which is heat sensitive (Nakamura et al., 2008). Following incubation at 95°C, sections were cooled for 5 min before being rinsed with PBS and blocked in 2% normal donkey serum diluted in PBS-Triton X-100 (0.1%) for 2 hours. Sections were then incubated overnight at 4°C with primary antibodies diluted in blocking buffer, washed 3 times in PBS for 10 min, and incubated with fluorescent secondary antibodies at room temperature for 2 h (Jackson ImmunoResearch). Sections were stained with 4′,6-diamidino-2-phenylindole (DAPI, Thermo Fisher Scientific) and mounted onto Superfrost slides (Thermo Fisher Scientific) with Aqua PolyMount (Polysciences).

The stained sections were imaged using a 40X oil objective on a SP5 or SP8 Leica confocal microscope, with a step size of 1-2 μm (X/Y pixel diameter = 0.28-0.38 μm). Cell counts were then performed on the imaged sections. To present normalized data in units of cells/DG, the area of the sampled SGZ was measured (SGZ length^∗^z stack depth). The calculated surface area was then scaled to an age-appropriate reference dataset from C57BL/6J mice of different ages (0.5, 1, 2, 6, 12 and 18 months) where the total SGZ surface area had been calculated. For measurements of fluorescence intensity the corrected total cellular fluorescence (CTCF) was calculated using the following formula: integrated density – (area of region of interest^∗^mean fluorescence of background). The data for fluorescence intensity is presented as arbitrary units in Figures 5 and 6. All cell counts were performed blind to the genotype being analyzed.

Throughout the analysis we identified NSCs as cells containing a radial GFAP-positive process linked to a SOX2-positive nucleus in the subgranular zone (SGZ). These cells were defined as quiescent or proliferating depending on expression of the cell-cycle marker Ki67. In the long-term self-renewal/persistence experiment (Figure 1K) we performed further validation that NSC identity was maintained long-term by ensuring the NSC was negative for the astrocytic marker S100β and therefore had not transformed into an astrocyte as postulated by the disposable stem cell model (Encinas et al., 2011; Martín-Suárez et al., 2019).

#### Tamoxifen treatment and administration of EdU

Tamoxifen solution (10-20 mg/mL) was prepared for intraperitoneal injections by dissolving the powder (Sigma-Aldrich) in a mix of 10% ethanol and 90% cornflower oil (Sigma-Aldrich). The dose and injection regime were selected according to the relative difficulty of inducing recombination of the floxed allele, whereas the route of administration changed from intraperitoneal injection to oral gavage dosing for experiments that lasted longer than 5-days, based on veterinary advice.

For experiments involving the *Huwe1*^*fl*^ lines, mice were injected with 80mg/kg of tamoxifen per day, whereas the daily dose delivered to *Ki67*^TD^ mice in Figure S1 and *H2B-GFP* mice in Figure 3, was 100mg/kg. Finally, for the Ki67^TD-NES^ mice used in the scRNA-seq experiments they were delivered tamoxifen via oral gavage for 6 consecutive days at 100mg/kg. In all experiments, control mice received tamoxifen at the same doses of experimental mice. The control mice were littermates that were wild-type for the floxed alleles and contained the *cre* transgene or were homozygous for the floxed alleles but negative for *cre*.

The labeling of cells progressing though S-phase was performed by either intraperitoneal injections of EdU (20 mg/kg) or through administration of EdU in the drinking water (0.2 mg/ml). For long-term EdU water administration, fresh solution was replaced at least every 56 hours. The sensitivity of EdU detection kits allowed for lower concentration of EdU to be used than in standard BrdU drinking water assays (typically 1mg/mL), and avoided obvious cellular toxicity (Young et al., 2013).

#### Calculation of observed/expected depletion rate

The observed NSC depletion rate was measured by first quantifying the reduction in NSC number between two time points (for example, between 0.5 months and 1 month of age). This was converted to the rate of change per day, which was normalized to the size of the NSC pool at the earlier time point (i.e., 0.5 months). This was repeated for all time points (2, 6, 12 and 18 months). The expected rate of depletion predicted by the disposable stem cell model was calculated by reducing the observed depletion date at the earliest instance (between 0.5-1 months of age) by the percentage reduction in the size of the proliferating NSC pool for all later time points (2, 6, 12 and 18 months).

#### Calculation of contribution to NSC proliferation

The contribution of dormant and resting NSCs to the proliferative (Ki67+) NSC pool is reported in Figures 2 and 6 from experiments in which mice received EdU in the drinking water for 2 weeks, prior to a 20 h chase period and culling. The number of proliferating (Ki67+) NSCs originating from the resting NSC pool was determined by multiplying the fraction of those Ki67+ NSCs that were EdU+, with the age-specific probability that the cell had at some point been in a resting state during the 2-week EdU period (determined by the fraction of total EdU+ NSCs that were Ki67–). This calculation assumes at minimum: the probability of a cell returning to quiescence is uniform throughout the 2-week EdU administration period, and that the likelihood of a cell returning to quiescence is the same after the 1^st^, 2^nd^ or *n*^th^ division. Finally, the fraction of proliferating NSCs originating from the dormant NSC pool was determined by subtracting the contribution of resting NSCs from the total.

#### H2B-GFP label dilution experiments

The dilution of the H2B-GFP nuclear label was used as a proxy to determine the number of self-renewing NSC divisions. Juvenile (0.5 months) and adult (5 months) cohorts were injected with tamoxifen (100mg/kg), each day for 5 days, to activate the creER^T2^ recombinase, recombine the *R26tTa* locus and induce H2B-GFP expression. The H2B-GFP label was allowed to build up for 14 days after tamoxifen administration, which was sufficient to produce bright and even labeling of NSC nuclei. After H2B-GFP labeling, the juvenile and adult cohorts were given 2 mg/ml doxycycline in drinking water, with EdU (0.2mg/mL) and sucrose (1% w/v) for 10 days, switching off H2B-GFP expression. This 10-day chase period corresponds to the approximate lifespan of an EdU+ NSC in 1-month old mice (Figure 1L). The solution was changed 3-times per week. After doxycycline treatment, juvenile mice (now 6-weeks of age) and adult mice (now 6 months of age) were immediately perfused and processed for immunostaining. As an additional control experiment, we later repeated the experiment in a new adult cohort using different build-up and chase periods. The length of the build-up period was increased to 25 days to allow for a more robust H2B-GFP labeling, considering that the expression of the *Rosa26* locus decreases with age. Importantly, we did not notice any difference in the sensitivity of H2B-GFP signal detection between the different build-up periods, suggesting that the extent of labeling was similar. Furthermore, the label-dilution period was adjusted to 30-days to match the approximate lifespan of an EdU+ NSC in adult mice, where it takes 30 days for more than 90% of the EdU+ NSC population to differentiate (Figure 1L). The experiment started when the mice were 4.5 months old to ensure they were 6-months old at the end of the chase period.

Sections were stained using antibodies against GFAP and SOX2 and were also stained for EdU and DAPI. Cell counts and GFP fluorescence intensity measurements were performed on the entire DG at different levels along the rostrocaudal axis. The GFP fluorescence intensity of EdU+ NSCs was compared to the mean GFP fluorescence intensity of 2-3 neighboring EdU– NSCs, to calculate a ratio. To minimize variability caused by staining or imaging artifacts, the EdU– cells that were selected for comparison had to be in the same X-Y image tile (291^∗^291 μm) and their center of mass within 8 z-planes (6.04 μm) of the EdU+ NSC. Segmentation of nuclei was performed using the Fiji plugin ‘3D Object Counter’ (Bolte and Cordelières, 2006), and the raw integrated density for each nucleus was used for the measurement output. Data points were excluded if the standard error of the mean of the average fluorescence intensity of EdU– NSCs was greater than 15% of the mean, as inclusion of this data detrimentally affected normalization.

All datasets from the juvenile and adult cohorts were pooled together to estimate the most probable number of clusters (modes) in the distribution, using the “modetest” function of the R package “multimode” (Ameijeiras-Alonso et al., 2018). Modes and anti-modes were then located by means of the “locmodes” function. and used to create bins that separated the values based on the number of cell divisions (n) that single EdU+ NSCs performed. The number of values falling in each bin was counted for each dataset. Therefore, we obtained the fraction of cells that divided n-times in each dataset.

#### Single-cell analysis of Ki67^TD-NES^ mice

##### Sample preparation

Each scRNA-seq experiment comprised 2-5 male and female Ki67^TD^; Nestin*-*GFP mice that had received tamoxifen via oral gavage for 6 consecutive days (100mg^∗^kg) and culled on the 8^th^ day. In total, 1 experiment was performed with 1-month-old mice, 3 experiments with 2-month old mice, and 3 experiments with 6-8-month-old mice (Table S2).

Mice were killed by cervical dislocation, their brains removed and the DG was microdissected (Hagihara et al., 2009; Walker and Kempermann, 2014). The dissected DG was then disassociated using the Neural Tissue dissociation kit (P) (Milteny Biotec) according to manufacturer’s instructions, with the following exceptions: a 37°C orbital shaker was used during the enzymatic digestions, and we used manual trituration with fire-polished pipettes to aid dissociation following the incubations with enzymatic mix 1 and 2. The dissociated cells were centrifuged and resuspended in 750 μL recovery media (0.5% PBS-BSA in DMEM/F-12 without phenol red and 1ug/ml DAPI. The cells were sorted on the MoFlo XDP (Bechman Coulter) using a 100μm nozzle with a pressure of 30 psi, and a sort efficiency of more than 80%. The events were first gated to remove debris (fsc-h versus ssc-h), to remove aggregates (typically pulse-width versus area then pulse-height versus area/width) and to remove dead cells (fsc-h versus DAPI fluorescence). Cells were then gated for tdTomato expression according to a control mouse that expressed Nestin-GFP alone; whereas the GFP gate was set according to the expected distribution of GFP fluorescence intensities based on prior control experiments. Cells were collected in 700 μl of recovery media in 1.5mL tubes, and spun down at 500G for 7 min at 4°C. After removing all but 50μl of the supernatant, the cells were then gently resuspended using a wide-bore pipette. The single-cell suspension (to a maximum of 10,000 cells) was then loaded into the 10x Chromium.

##### Sequencing and mapping

We prepared one library for each of the 8 samples across the 7 experimental days. The libraries for 2-month-old mice were prepared with 10x Genomics Chemistry, Single Cell 3′ version 2, while the 1-month and 6-month libraries were prepared with version 3 (Table S2). After sequencing, cellRanger count (Ver. 3.0.2) was used to map the FASTQ files to our custom mouse genome (mm10-3.0.0), which contained 4 functional elements. The first element was the GFP coding sequence to detect transcription of Nestin-GFP that appears as “eGFP” in the final data matrix. The second, was the 5′ floxed sequence of the *tdTomato* allele that detects transcription of the intact *tdTomato* locus and appears as “tdTomatoLoxP” in the final data matrix. Finally, we added 3′ regions of the *tdTomato* locus that are transcribed upon recombination, which encode the *woodchuck hepatitis virus post-transcriptional regulatory element* (appears as “WPRE” in the final data matrix) and the *bovine growth hormone* polyA signal (appears as “bGHpolyA” in the final data matrix). The coding sequence of the *tdTomato* gene was also originally included in the custom genome as a 5^th^ functional custom element, however very few transcripts mapped to this region, and the origins of these transcripts (intact versus recombined *tdTomato* locus) were unclear as the *tdTomato* coding sequence is directly downstream of the 5′ polyA signal but more than 600 base pairs upstream of the 3′ polyA signal. As such the data was remapped with this feature disabled. The disabling of this genomic region is marked by the empty feature “tdTomatoCDS” in the final data matrix. The custom genome was made with the cellRanger mkref command.

##### Seurat analysis: Quality control

The cellRanger aggr command was used to merge the count files of the 8 individual libraries without normalization. The merged matrix was then read into Seurat (Ver. 3.1.2) for analysis (Stuart et al., 2019). GEM beads were kept for further analysis if they contained more than 500 genes and fewer than 10% mitochondrial reads in order to remove GEM beads that contained only background signal or a dead cell. Doublets/multiplets were then removed based on the expression of more than 1 marker gene per GEM bead (e.g., co-expression of the astrocytic marker *Aldoc* with the oligodendrocyte marker *Mog*). In total, 24,203 of 26,036 (92.95%) passed these quality control steps. Later, during the subsetting/re-clustering of the data further small doublet clusters appeared, which were identified and removed based on the lack of specific marker expression (Briggs et al., 2018).

##### Seurat analysis: Distinguishing G_1_ from G_0_

We then added metadata that marked each cell for tdTomato expression and cell-cycle status. Cells were identified as tdTomato+ if they contained < 4 reads of the intact *tdTomato* locus, and more than > 1 read of the recombined *tdTomato* locus based on ground-truth testing of our first dataset, in which we sequenced the tdTomato+ and tdTomato– populations separately (Figure S3). We also identified whether a cell was in G_0_ or proliferating using ground-truth testing. We isolated bona-fide S-phase cells through the CellCycleScoring function in Seurat, and bona-fide G_0_-phase NSCs based on UMAP position and plotted the expression in these cells of genes known to be highly and stably expressed across both *G*_*1*_*-* and *S*-phases (i.e., *Mcm2-7*, *Ccne1/2*, and *Pcna*) (Braun and Breeden, 2007).We binarized each cell as positive or negative for each marker and plotted every cell according to their index score (range of possible values 0-9). These plots revealed a clear separation of two cellular populations which we used to distinguish in the remainder of the dataset, tdTomato+ cells that were in G_1_ (that we marked as proliferating NSCs) or in G_0_ (resting NSCs). Specifically, cells in which we detected > 1 index gene were scored as in G_1_, while all other cells were considered quiescent/post-mitotic. Cells sequenced with Version 3 of the 10x Chemistry had on average twice the number of genes detected, so the thresholds for tdTomato and cell-cycle scoring were doubled.

##### Seurat analysis: Data visualization

The merged Seurat object was split according to library/experimental day and the data transformed using the SCTransform function. The 5,000 most variable genes and anchors across the 8 datasets were then identified and the data integrated using the default parameters. The integrated dataset was then visualized using UMAP (Becht et al., 2018). Elbow plots were used to determine the number of significant principal components. Following cluster identification with known marker genes, we extracted all NSCs and IPCs and re-clustered these cells using the same workflow. We iterated this process a further time to then extract only NSCs, this time splitting and integrating the Seurat object according to 10x Chemistry (V2 or V3), as this best eliminated technical variation without removing biological signal (assessed by clustering of replicates). We appended metadata classifying these cells as dormant NSCs (quiescent and tdTomato–), resting NSCs (quiescent and tdTomato+) or proliferating NSCs (proliferating and tdTomato+/–). The final dataset contained 2,947 cells which we processed for differential gene expression and pseudotime ordering.

##### Pseudotime ordering

To construct a pseudotime ordering of NSCs we used the trajectory inference tool Slingshot (Street et al., 2018) (Ver. 1.2.0) and UMAP dimensionality reduction. The curve generated by Slingshot was used to plot the pseudotime position of each cell between conditions and to generate a set of genes statistically associated (p < 0.01) with pseudotime progression. For the reconstruction of the pseudotime trajectory from Shin et al. (2015) we read in the normalized counts (TPM) from GSE71485 into Seurat. Next PCA analysis was performed using the 5 top components and the data was then visualized using UMAP. We excluded a cluster of cells that lacked significant expression of *Hopx* that largely corresponded to contaminating oligodendrocytes, as reported in the original study. We compared the set of genes that were statistically associated with pseudotime with our dataset.

### Quantification and statistical analysis

#### Single-cell data

Differential gene expression analysis was performed using the FindMarkers function in Seurat using the Pearson residuals located in the “scale.data” slot of the SCT assay using Student’s t test (Hafemeister and Satija, 2019). No minimum log fold change threshold was enforced; however, to be included in the final analysis, all genes had to be expressed by a minimum of 20% of cells in at least one of the two conditions being compared and were considered as statistically significant only if they had an FDR adjusted *P*-value of < 0.05. For visualisation of gene expression differences between groups the normalised data from the "RNA" assay are presented as violin plots throughout the manuscript.

#### Gene Ontology

The Gene Ontology analysis was performed on statistically significant genes using the clusterProfiler package (3.12.0) (Yu et al., 2012) with the onotology term “BP” for biological process. This analysis was performed separately on 1) all statistically significant genes together, 2) upregulated genes only and 3) downregulated genes only. The results of these analyses are reported in Tables S3, S4, and S5.

#### Statistical analysis of cell counts

The statistical testing approach were specified prior to data collection and implemented using Graphpad Prism (version 8.0) or in R (3.6.2). Two-tailed unpaired Student’s t tests or Mann-Whitney U tests were performed when comparing two groups. For experiments involving one or two independent variables, one or two-way ANOVA was performed, respectively. Any significant main effect detected by ANOVA was followed by multiple t tests when required using a pooled estimate of variance where appropriate, and significance was corrected for using the Holm-Sidak method in Prism 8.0 (Graphpad). Fisher’s exact test was used in Figure 3. Details of statistical tests for specific experiments are found in figure legends.

## Supporting citations

The following references appear in the Supplemental Information: Brandt et al., 2012, Burnham and Anderson, 2002, Marciniak-Czochra et al., 2009, Stiehl and Marciniak-Czochra, 2011, Stiehl and Marciniak-Czochra, 2017, Ziebell et al., 2014.

## Supplemental information

Document S1. Figures S1–S7 and Tables S1 and S2

Table S3. Differential gene expression analysis of resting versus dormant hippocampal NSCs. related to Figure 5

Table S4. Differential gene expression analysis of resting versus proliferating hippocampal NSCs, related to Figure 5

Table S5. Differential gene expression analysis of quiescent hippocampal NSCs from 6-month-old versus 1-month-old mice, related to Figures 6 and 7

Data S1. Detailed description of the mathematical model, related to Figures 1, 2, 3, and S2 and Table S1

Document S2. Article plus supplemental information
