# Supplementary material for: Coordinated changes in cellular behavior ensure the lifelong maintenance of the hippocampal stem cell population
Source: Cell Stem Cell. 2021 May 6;28(5):863–876.e6. doi: 10.1016/j.stem.2021.01.003 (PMC8110946; doi:10.1016/j.stem.2021.01.003)
Supplement: Document S1. Figures S1–S7 and Tables S1 and S2 [file mmc1.pdf]

Cell Stem Cell, Volume *28*

Supplemental Information

Coordinated changes in cellular behavior ensure the lifelong maintenance of the hippocampal stem cell population

Lachlan Harris, Piero Rigo, Thomas Stiehl, Zachary B. Gaber, Sophie H.L. Austin, Maria del Mar Masdeu, Amelia Edwards, Noelia Urbán, Anna Marciniak-Czochra, and François Guillemot

SUPPLEMENTAL ITEMS

Figure S1

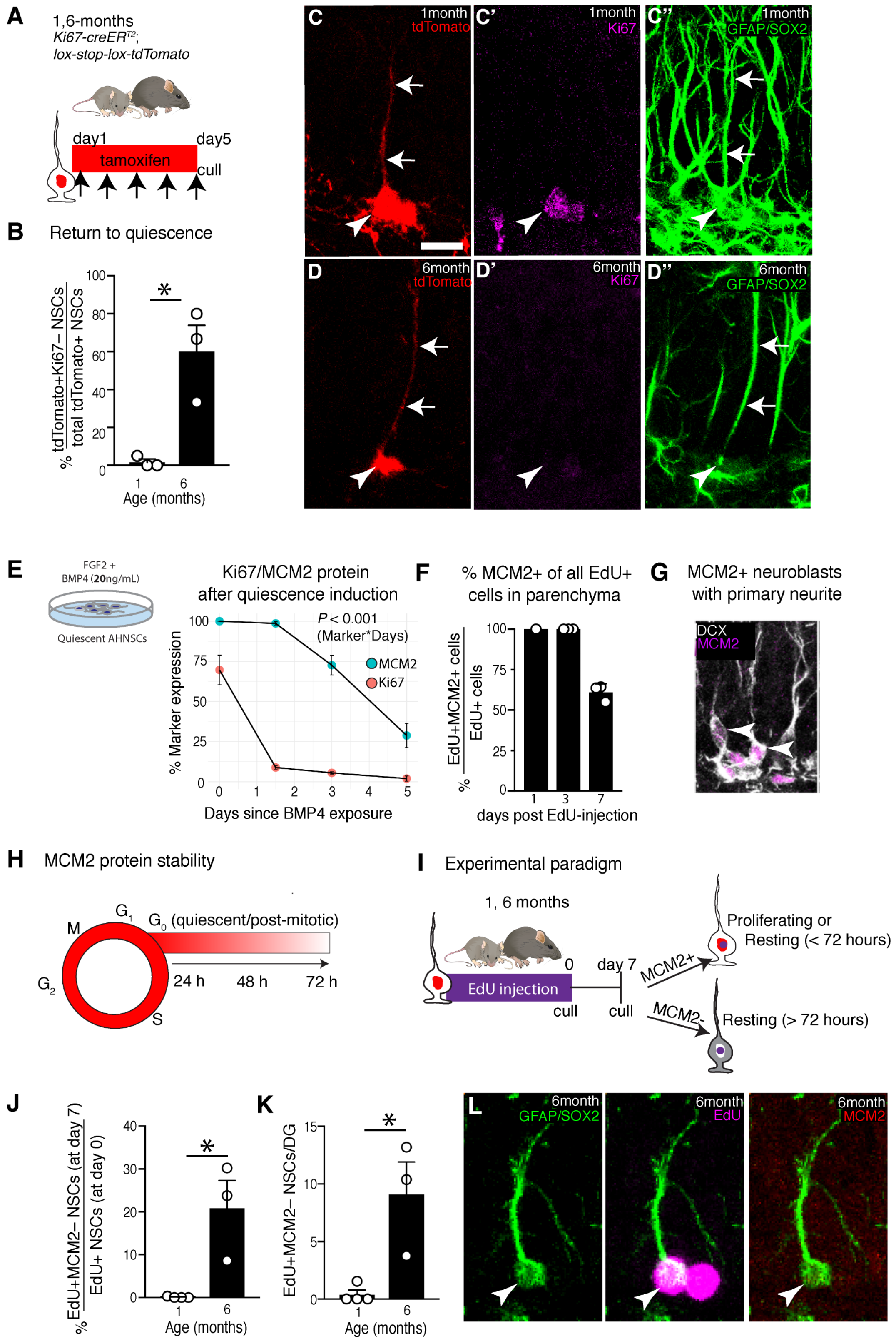

Figure S1, related to Figure 1: Genetic lineage tracing of proliferating NSCs and the absence of MCM2 protein confirm an increasing rate of return to quiescence with age.

(**A**) Upon tamoxifen administration, proliferating NSCs and their progeny, including resting NSCs, are indelibly labelled with tdTomato fluorescence in Ki67^{TD} mice. (**B**, **C**) In 1-month-old mice culled immediately after tamoxifen treatment, tdTomato+ NSCs remain proliferating (Ki67+). whereas in 6-month-old animals (**B**, **D**) a substantial proportion returns to quiescence (Ki67–). In (**C**, **D**) arrowheads indicate NSC cell body, arrows indicate the radial tdTomato+ process. (**E-G**) The absence of MCM2 protein, due to its expression throughout the entire cell-cycle (including G_1) and its extended perdurance, identifies cells that have returned to quiescence for prolonged periods. (**E**) The *in vitro* stability of MCM2 protein is demonstrated by its slow decay (relative to Ki67) in hippocampal-derived NSPC cultures after inducing quiescence through the addition of BMP4. (**F**) The *in vivo* stability of MCM2 protein was measured by injecting 4-6 week old mice with EdU, culling the mice 1, 3 and 7 days later and assessing EdU+ cells in the hippocampal parenchyma for MCM2 protein. MCM2 is detected for at least 3 days after a cell has exited the cell-cycle. (**G**) For example, MCM2 is detected in mature neuroblasts with a primary neurite that extends into the molecular layer (arrowheads). (**H**) Summary schematic of MCM2 protein expression during cell-cycle progression and after cell-cycle exit. (**I**) The frequency in which EdU-incorporating hippocampal NSCs in 1- and 6-month-old mice return to quiescence is determined by measuring the fraction of EdU+ NSCs that were MCM2– one-week after EdU injections (single injection at 9am and 5pm). (**J**) 20.9% of EdU+ NSCs initially labelled at day 0 were MCM2– at day 7 in 6-month old mice, which was approximately 200-fold higher rate than in 1-month old mice. (**K**) Similarly, the absolute number of EdU+MCM2– cells in 6-month old mice was 23-fold higher than in the younger cohort. (**L**) Image of an EdU+MCM2– NSC in 6-month old mice 7 days post EdU-labelling.

Graphs in **B, E, F, J and K** show mean ± s.e.m, dots represent individual mice. In **E** the experiment was replicated at least twice per timepoint. Statistics: t-test in **B, J and K** and two-way ANOVA in **E** (interaction effect of day*marker). Scale bar (in **C**): **C**, **D** = 15 μm; **G** = 25 μm and **L** = 7 μm. *$P<0.05$.

Figure S2

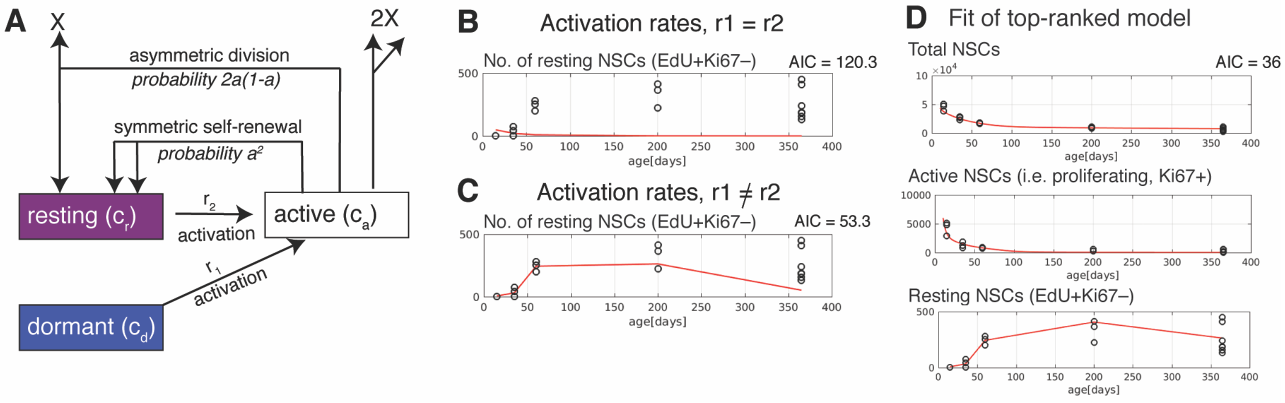
A
X
2X
asymmetric division
probability 2a(1-a)
symmetric self-renewal
probability a²
resting (cr)
r2
activation
active (ca)
r1
activation
dormant (cd)
B
Activation rates, r1 = r2
No. of resting NSCs (EdU+Ki67–)
AIC = 120.3
age[days]
C
Activation rates, r1 ≠ r2
No. of resting NSCs (EdU+Ki67–)
AIC = 53.3
age[days]
D
Fit of top-ranked model
Total NSCs
AIC = 36
age[days]
Active NSCs (i.e. proliferating, Ki67+)
age[days]
Resting NSCs (EdU+Ki67–)
age[days]

Figure S2, related to Figure 1-3, Table S1 and data file 1: Mathematical modelling of time-dependent changes

(**A**) Mathematical model of age-related changes of NSC numbers in the mouse hippocampus that distinguishes between different NSC subpopulations, namely active (i.e. proliferating Ki67+) NSCs, and quiescent NSCs (identified by absence of Ki67 expression). The quiescent NSC population is further subdivided into dormant NSCs, i.e. NSCs that not have not activated since establishment of the niche at postnatal day 14 and resting NSCs, i.e. quiescent NSC that have already activated (proliferated) since postnatal day 14. The model describes the time evolution of active (proliferating), dormant and resting NSC counts after postnatal day 14. The time-dependent parameters explored in the model included the activation rates of dormant (r_1) and resting (r_2) cells, self-renewal (α) and proliferation rate/cell-cycle length (p). Non-stem cells in the model are denoted by X. (**B**, **C**) Fitting of different models to the data favoured the scenario where dormant and resting NSC activation rates decreased with time but were higher for resting than for dormant NSCs, as shown in the fit of these models to the number of resting NSCs against time. (**D**) Fit of top model (lowest AIC) to number of dormant NSCs, active NSCs and resting NSCs. AIC = Akaike Information Criterion

Figure S3

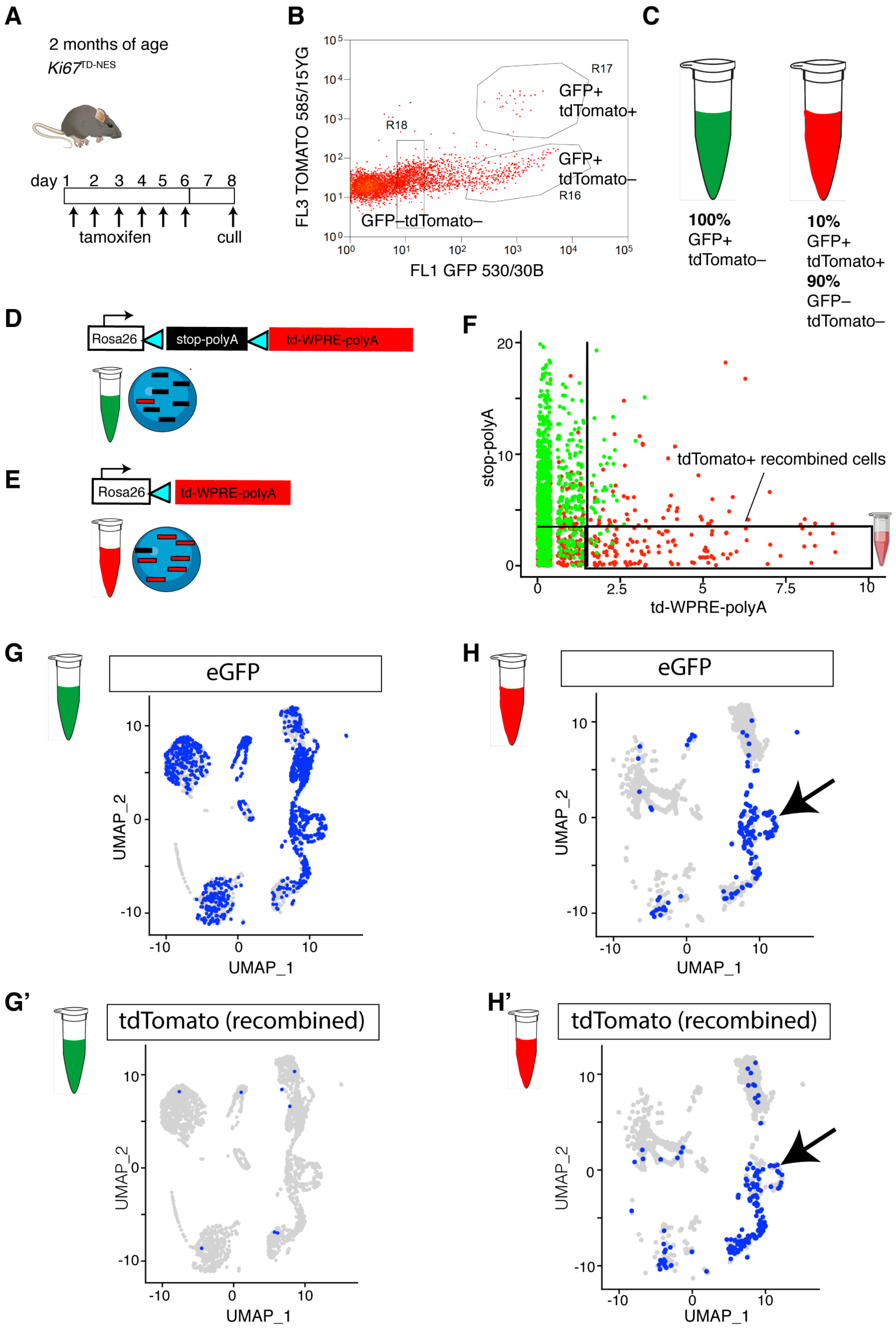
A
2 months of age
Ki67TD-NES
day 1 2 3 4 5 6 7 8
tamoxifen
cull
B
FL3 TOMATO 585/15YG
FL1 GFP 530/30B
R17
GFP+ tdTomato+
R18
GFP+ tdTomato–
R16
GFP–tdTomato–
C
100% GFP+ tdTomato–
10% GFP+ tdTomato+
90% GFP– tdTomato–
D
Rosa26
stop-polyA
td-WPRE-polyA
E
Rosa26
td-WPRE-polyA
F
stop-polyA
td-WPRE-polyA
tdTomato+ recombined cells
G
eGFP
UMAP_2
UMAP_1
H
eGFP
UMAP_2
UMAP_1
G'
tdTomato (recombined)
UMAP_2
UMAP_1
H'
tdTomato (recombined)
UMAP_2
UMAP_1

Figure S3, related to Figure 4: Post-sequencing identification of cells that have undergone *cre*-mediated recombination in Ki67^{TD-NES} mice.

(**A**) Schematic of pilot experiment to determine the efficacy of identifying tdTomato+ cells after sequencing in *Ki67-creER^{T2}*; *lox-STOP-lox-tdTomato*; *Nestin-GFP* mice (Ki67^{TD-NES}). (**B**, **C**) GFP+tdTomato– cells and GFP+tdTomato+ cells were collected in separate tubes. Because GFP+ tdTomato+ cells were rare (~1,000 in this experiment) they were also mixed with 9,000 GFP– tdTomato– cells to ensure there was a minimum of 10,000 cells to centrifuge and recover before loading into the 10x Chromium. (**D**, **E**) The two tubes were loaded into the 10x Chromium. (**D**) Recombined cells should have low/absent expression of the floxed "*td-stop*::polyA" transcript, whereas (**E**) they should have higher expression of the downstream "*td::WPRE::bGH*::polyA" transcript. (**F**) Scatterplot of intact (y-axis) *versus* recombined transcripts (x-axis) demonstrates that cells with a recombined signature almost entirely come from the GFP+ tdTomato+ tube. (**G**) UMAP plots of cells from GFP+tdTomato– tube demonstrate almost all cells are GFP+ and tdTomato– (**H**) UMAP plots of cells from GFP+ tdTomato+/GFP– tdTomato– tube shows that in contrast, approximately 90% of the cells are negative for both markers, corresponding to the GFP–tdTomato– cells that were loaded to weight the GFP+ tdTomato+ sample, whereas the remaining cells have high expression of GFP and tdTomato and localise to proliferative cells, i.e. intermediate neuronal progenitors and neuroblast clusters (arrow).

Figure S4

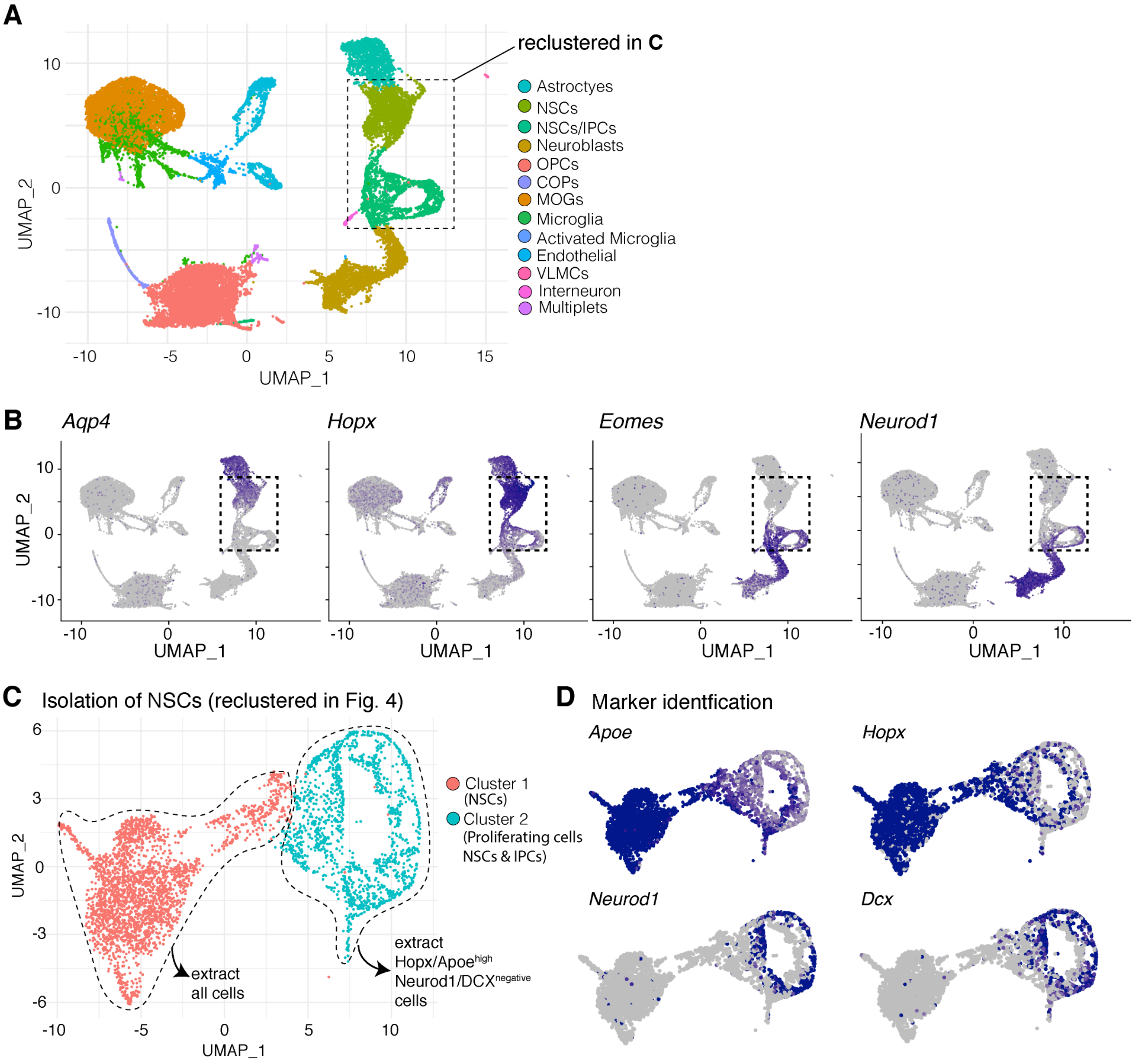
A
reclustered in C
Astroctyes
NSCs
NSCs/IPCs
Neuroblasts
OPCs
COPs
MOGs
Microglia
Activated Microglia
Endothelial
VLMCs
Interneuron
Multiplets
UMAP_1
UMAP_2
B
Aqp4
Hopx
Eomes
Neurod1
C
Isolation of NSCs (reclustered in Fig. 4)
Cluster 1 (NSCs)
Cluster 2 (Proliferating cells NSCs & IPCs)
extract all cells
extract Hopx/Apoe^high Neurod1/DCX^negative cells
D
Marker identfication
Apoe
Hopx
Neurod1
Dcx

Figure S4, related to Figure 4: Identification and isolation of NSCs from merged dataset.

(**A**) UMAP plot of cell clusters from merged dataset of 8 libraries (different ages and replicates) with their cellular identity indicated with the colour key. (**B)** The NSC cluster was identified based on an *Aqp4*-low and *Hopx*-high expression profile. The IPC cluster was identified based on an *Eomes*-high and *Neurod1*-high expression profile. Dashed boxes in **A** and **B** indicate the selection and isolation of NSC and IPC clusters for re-clustering in **C**. (**C**) UMAP plot displaying NSCs and IPCs from merged dataset. Cluster 1 was comprised entirely of NSCs and cluster 2 was comprised of proliferating cells (IPCs and some NSCs). All cells in cluster 1 were extracted as NSCs for further analysis, (**D**) whereas only cells that had high *Hopx*/*Apoe* expression and were negative for both IPC *mRNA* markers *Neurod1* and *Dcx* were extracted as NSCs from cluster 2. These extracted cells were then re-clustered and are presented in Figure 4D-J.

Figure S5

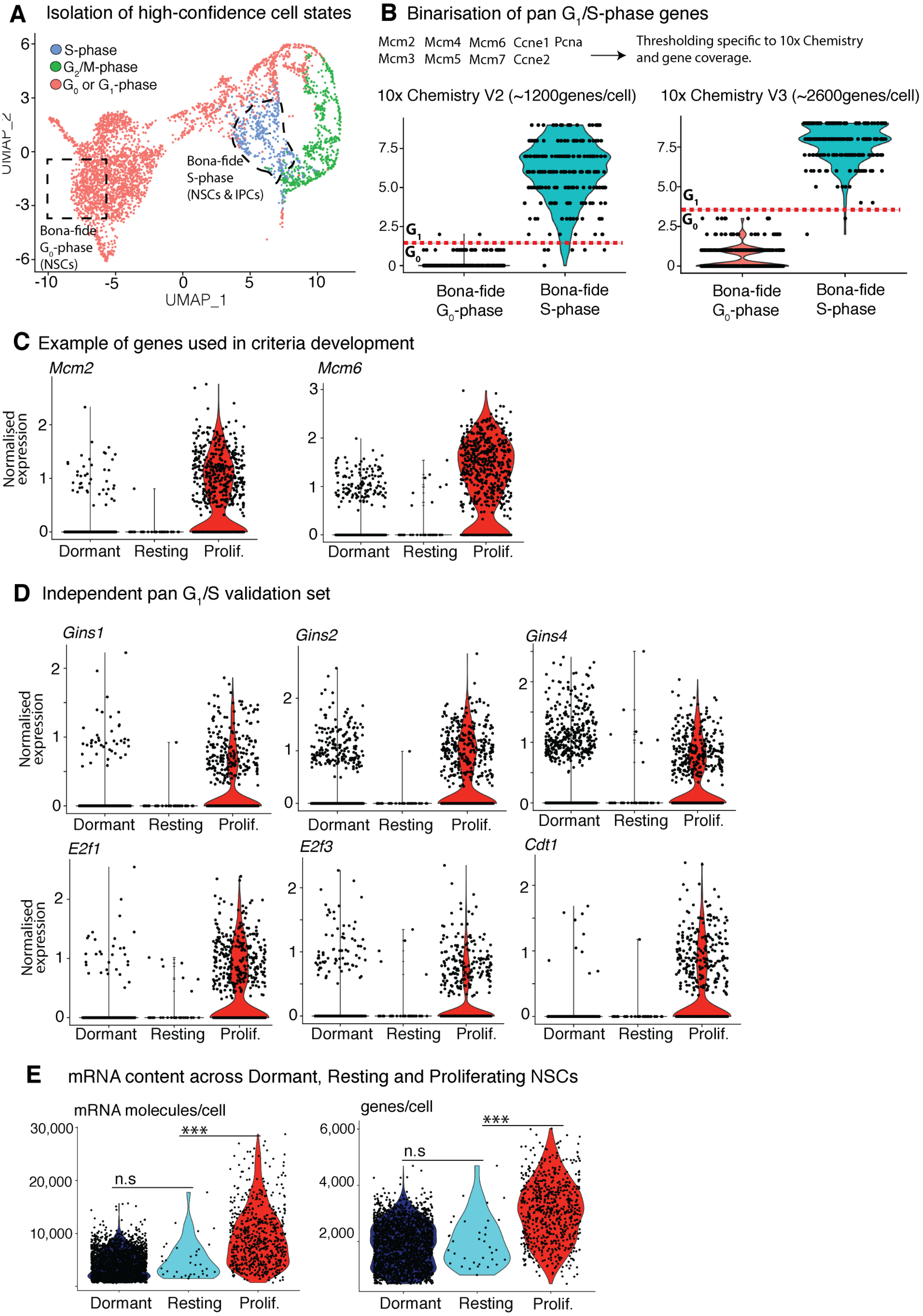

A Isolation of high-confidence cell states
S-phase
G2/M-phase
G0 or G1-phase
Bona-fide S-phase (NSCs & IPCs)
Bona-fide G0-phase (NSCs)
UMAP_1
UMAP_2
B Binarisation of pan G1/S-phase genes
Mcm2 Mcm4 Mcm6 Ccne1 Pcna
Mcm3 Mcm5 Mcm7 Ccne2
Thresholding specific to 10x Chemistry and gene coverage.
10x Chemistry V2 (~1200genes/cell)
10x Chemistry V3 (~2600genes/cell)
G1
G0
Bona-fide G0-phase
Bona-fide S-phase
C Example of genes used in criteria development
Mcm2
Mcm6
Normalised expression
Dormant Resting Prolif.
D Independent pan G1/S validation set
Gins1
Gins2
Gins4
E2f1
E2f3
Cdt1
E mRNA content across Dormant, Resting and Proliferating NSCs
mRNA molecules/cell
genes/cell
n.s
***

Figure S5, related to Figure 5: Thresholding criteria to distinguish G_0 from G_1 phase.
(**A**) UMAP plot showing NSC and proliferating cell (NSC/IPC) clusters. For ground-truth testing bona-fide S-phase NSC/IPCs were identified through the CellCycleScoring function in Seurat, whereas bona-fide G_0-phase NSCs were isolated based on position in UMAP plot corresponding to deep quiescence. (**B**) Genes that are highly and stably expressed in both G_1- and S-phase (e.g. *Mcm2* and *Mcm6*) were binarized and added together, to generate an index. Violin plots of this index showed a clear hourglass separation between G_0 and S-phase cells, which allowed for thresholds (separate thresholds for each version of the 10x kit) to be chosen to distinguish G_0 and G_1 in the remainder of the dataset. Cells above the threshold were designated as G_1 and therefore as proliferating cells alongside S- and G_2/M-phase cells. (**C**) Plots of *Mcm2* and *Mcm6* expression, two genes used in the generation of the thresholding index. (**D**) This thresholding was also tested in an independent set of G_1- and S-phase genes, which showed that resting NSCs do not express significantly higher levels of these genes than dormant NSCs, and much lower levels than proliferating NSCs. (**E**) Attesting to the quiescent state of resting NSCs they also have half the level of mRNA as proliferating NSCs, comparable to dormant NSCs. ***P<0.001.

Figure S6

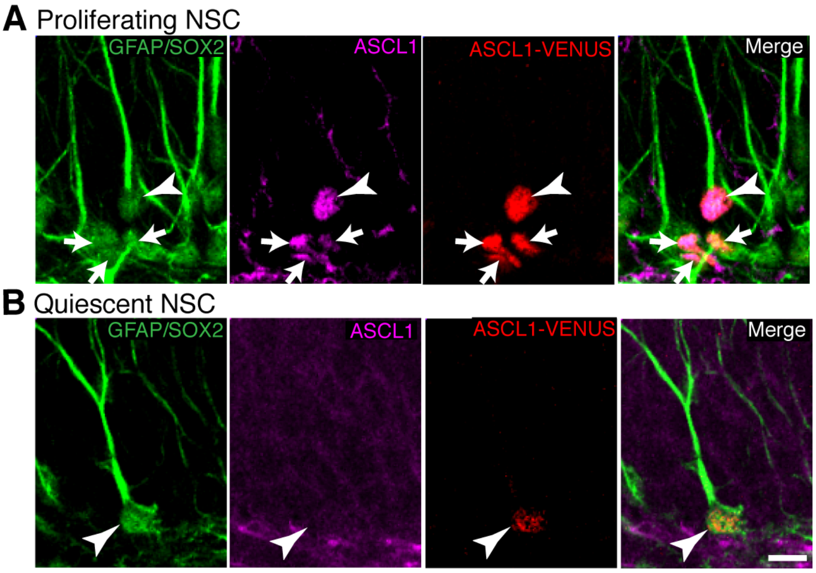
A Proliferating NSC
GFAP/SOX2
ASCL1
ASCL1-VENUS
Merge
B Quiescent NSC
GFAP/SOX2
ASCL1
ASCL1-VENUS
Merge

Figure S6, related to Figure 6: Increased detection sensitivity of ASCL1 protein using Ascl1-Venus mice and anti-GFP antibodies.

(**A, B**) 3-week-old Ascl1-Venus mice, expressing a Ascl1-Venus fusion protein were stained for neural stem cell markers (GFAP and SOX2) as well as ASCL1 using both an anti-ASCL1 antibody and an anti-Venus/GFP antibody. (**A**) In proliferating NSCs (proliferating status indicated by penumbra of SOX2+GFAP– IPCs surrounding the NSC), ASCL1 protein was expressed at high levels and produced a strong signal using both antibodies. **(B)** In contrast, ASCL1 protein was expressed at very low levels in quiescent NSCs and could only be detected with the anti-Venus/GFP antibody. Scale bar (in **B**): **A**, **B** is 10 μm.

Figure S7

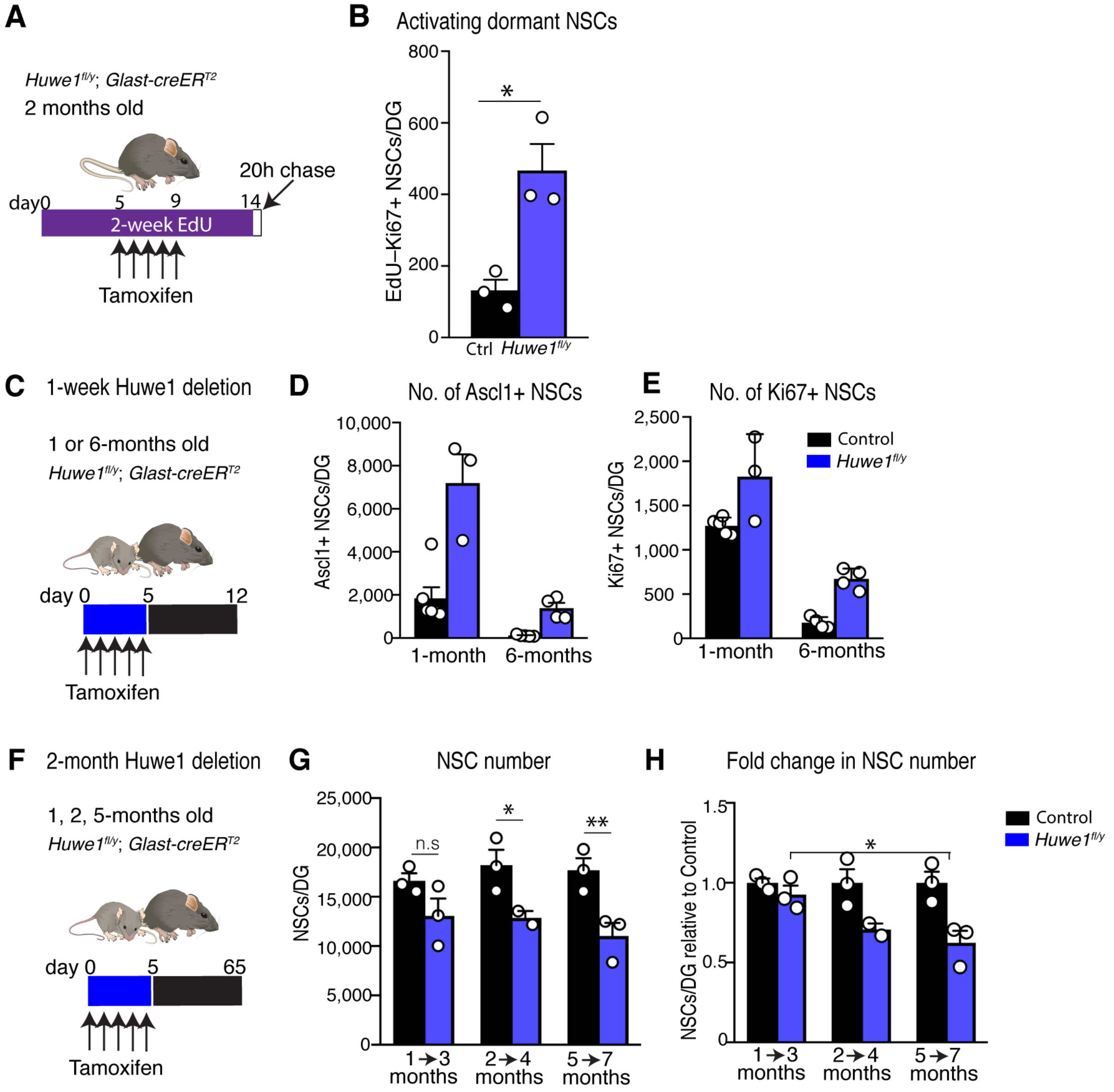
A
Huwe1fl/y; Glast-creERT2
2 months old
20h chase
day0
5
9
14
2-week EdU
Tamoxifen
B
Activating dormant NSCs
EdU–Ki67+ NSCs/DG
800
600
400
200
0
*
Ctrl
Huwe1fl/y
C
1-week Huwe1 deletion
1 or 6-months old
Huwe1fl/y; Glast-creERT2
day 0
5
12
Tamoxifen
D
No. of Ascl1+ NSCs
Ascl1+ NSCs/DG
10,000
8,000
6,000
4,000
2,000
0
1-month
6-months
E
No. of Ki67+ NSCs
Ki67+ NSCs/DG
2,500
2,000
1,500
1,000
500
0
Control
Huwe1fl/y
1-month
6-months
F
2-month Huwe1 deletion
1, 2, 5-months old
Huwe1fl/y; Glast-creERT2
day 0
5
65
Tamoxifen
G
NSC number
NSCs/DG
25,000
20,000
15,000
10,000
5,000
0
n.s
*
**
1→3 months
2→4 months
5→7 months
H
Fold change in NSC number
NSCs/DG relative to Control
1.5
1.0
0.5
0
*
Control
Huwe1fl/y
1→3 months
2→4 months
5→7 months

Figure S7, related to Figure 7*:* ***Huwe1*** **suppresses the activation of dormant NSCs and increased activity of *Huwe1* with age.**

(**A**) *Huwe1*$^{fl/y}$; *Glast-creER*^{T2} mice (*Huwe1*$^{fl/y}$) and controls were given EdU in drinking water for 2-weeks, during this time they also received tamoxifen. (**B**) Dormant NSCs (EdU–) in *Huwe1*$^{fl/y}$ mice showed increased activation during the 20 h chase period (EdU– Ki67+) compared with controls. (**C**) 1- and 6-month-old *Huwe1*$^{fl/y}$ and control mice were culled 1-week after receiving tamoxifen. (**D, E**) While the number of ASCL1+ and Ki67+ NSCs were increased upon *Huwe1* deletion at both ages, the effect was much larger in 6-month-old mice relative to age matched controls (see Figure 7 for graphs showing fold-change). (**F**) Huwe1 was deleted in hippocampal neural stem cells at 1, 2 and 5-months of age. Mice were then culled 2-months later. (**G**) Deletion of Huwe1 at 2 and 5 months but not at 1 month, led to a reduction in the number of NSCs relative to age-matched controls. (**H**) The fold change reduction in NSC number was significantly larger at the 5-month timepoint than the 1-month timepoint.

Graphs represent mean ± s.e.m. Dots represent the mean of individual mice. Statistics: t-test in **B**, **G** and **H**. **P*<0.05, ***P*<0.01.

Table S1, related to Figure 3 and STAR methods: List of main mathematical models investigated.

AIC	Decreasing activation rates	Equal activation rates ($r_1 = r_2$)	Increasing self-renewal	Positive lower-bound on activation rates	Increasing cell-cycle length (*p*)	No self-renewal ($a = 0$)
**36**	Yes	No	No	Yes	No	No
**36.3**	Yes	No	Yes	Yes	Yes	No
**37.9**	Yes	No	No	Yes	Yes	No
**41.4**	Yes	No	Yes	Yes	No	No
**53.3**	Yes	No	No	No	No	No
**56.1**	Yes	No	Yes	No	No	No
**120.3**	Yes	Yes	No	No	No	No
**125.3**	Yes	No	No	Yes	No	Yes
**152**	No	No	No	No	Yes	No

Table S2, related to Figure 4: List of single-cell RNA sequencing experiments performed in Ki67$^{TD\text{-}NES}$ mice.

Date	Sample	Exp	Age	GFP+ or GFP+tdT+	Cell no.	10x 3' Chemistry	Reads/cell	Genes/cell	GEM suffix
05-Sep-18	1	1	2 mo	GFP+tdT+	1,608	V2	66,023	1,248	-2
05-Sep-18	2	1	2 mo	GFP+	2,776	V2	37,832	1,497	-3
16-Apr-19	3	2	2 mo	combined	4,461	V2	34,309	1,088	-4
17-Apr-19	4	3	2 mo	combined	1,955	V2	37,975	1,305	-5
29-May-19	5	4	6 mo	combined	2,768	V3	55,514	2,013	-6
17-Sep-19	6	5	6 mo	combined	3,524	V3	93, 049	2,652	-7
19-Sep-19	7	6	6 mo	combined	5,412	V3	114, 637	3,253	-8
18-Oct-19	8	7	1 mo	combined	3,532	V3	56, 457	2,710	-1
