# Supplementary material for: Coordinated changes in cellular behavior ensure the lifelong maintenance of the hippocampal stem cell population
Source: Cell Stem Cell. 2021 May 6;28(5):863–876.e6. doi: 10.1016/j.stem.2021.01.003 (PMC8110946; doi:10.1016/j.stem.2021.01.003)
Supplement: Data S1. Detailed description of the mathematical model, related to Figures 1, 2, 3, and S2 and Table S1 [file mmc5.pdf]

Mathematical Modeling

Contents

1 Model description **2**
1.1 Purpose of the model 2
1.2 Data and experimental setup 3
1.3 Model derivation 3
1.4 Model of the labeling experiments 5
1.4.1 Labeling phase 5
1.4.2 Chase period 7
1.5 Full model 8

2 Model simulations **9**

3 Model quantification **9**

4 Model selection **9**

5 Age-related change of cell parameters **10**

6 Fitting active and total NSC dynamics **10**
6.1 Identical activation rates for dormant and resting NSCs 10
6.2 Different activation rates for dormant and resting NSCs 11

7 Fitting total, active and resting NSC dynamics **12**
7.1 Identical activation rate for dormant and resting NSCs 12
7.2 Different activation rates for dormant and resting NSCs 13
7.3 Different activation rates for dormant and resting NSCs and age dependent self-renewal 14
7.4 Model with non-zero activation for large time 16

7.4.1 Constant self-renewal 16
7.4.2 Age-dependent self-renewal 17
7.5 Age-dependent proliferation 19
7.6 Age-dependent self-renewal and proliferation 20

8 Study of specific scenarios **22**
8.1 Proliferation rate as only age-dependent cell parameter 22
8.2 Scenarios without self-renewal 23

9 Model with different EdU administration periods **24**

10 Model without return to quiescence **26**
10.1 Labeling phase 28
10.2 Chase period 29
10.3 Fitting 30
10.3.1 Maximal number of 5 divisions 31
10.3.2 Maximal number of 10 divisions 32
10.3.3 Maximal number of 50 divisions 33
10.4 Conclusion 33

1 Model description

1.1 Purpose of the model

The aim of the model is to understand age-related changes of neural stem cell (NSC) numbers in the mouse hippocampus. We distinguish between different NSC subpopulations, namely actively proliferating NSCs (identified by expression of Ki67 and referred to as proliferating NSCs in the main text) and quiescent NSCs (identified by absence of Ki67 expression). The quiescent NSC population is further subdivided in dormant NSCs, i.e., NSCs that have never been activated since establishment of the niche at postnatal day 14 and resting NSCs, i.e., quiescent NSCs that have already been activated since establishment of the niche at postnatal day 14. The model describes the time evolution of active, dormant and resting NSC counts after postnatal day 14.

1.2 Data and experimental setup

Total, active and resting NSC counts were experimentally determined in mice aged 15, 35, 60, 200 and 365 days. For the measurements the animals were sacrificed such that each data point comes from a different individual.

NSCs were counted in brain sections. Hippocampal NSCs were defined as cells containing a radial GFAP-positive process linked to a SOX2-positive nucleus in the subgranular zone. Ki67 was used as a marker to distinguish between active (Ki67 positive) and quiescent (Ki67 negative) stem cells.

The resting NSC counts were approximated by the following experimental procedure: Mice were exposed to the thymidine analogue EdU for 14 days followed by an EdU free period of 20 hours (referred to as "chase"). During DNA repliction EdU is incorporated into the DNA. After the chase period the animals were sacrificed. Resting cells were defined as EdU positive Ki67 negative cells, i.e., cells that have divided during the EdU exposure but have returned to a quiescent state at the time of cell counting.

1.3 Model derivation

The model describes time evolution of active, resting and dormant NSCs. As stated above, NSCs that have never divided since postnatal day 14 are denoted as dormant. Quiescent NSCs that have divided since postnatal day 14 are denoted as resting NSCs. Proliferating stem cells are denoted as active. The model considers the following processes

- Dormant NSCs are activated at the rate r_1.
- Resting NSCs activated at the rate r_2.
- Active stem cells divide at the rate p.
- Upon division an active NSC gives rise to two progeny. With probability a a progeny cell is again a stem cell (referred to as self-renewal), with probability $(1-a)$ it is an intermediate neuronal progenitor cell (referred to as differentiation). The probability a is referred to as *self-renewal probability* or *fraction of self-renewal* (Stiehl and Marciniak-Czochra, 2011, 2017; Marciniak-Czochra et al., 2009).
- In agreement with our previous work and with experimental data (Ziebell et al., 2018; Kalamakis et al., 2019) we assume that NSCs originating from division become quiescent.

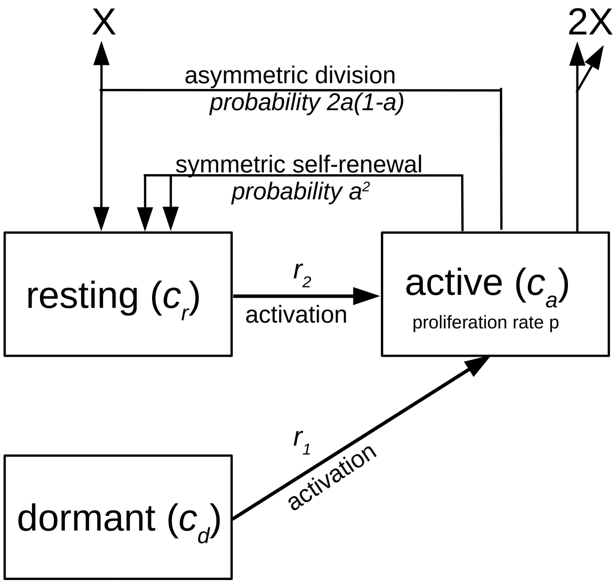

Figure 1: NSC model. The scheme shows processes described by system (1). Non-stem cells are denoted by X.

The model is summarized in Figure 1. It is an extension of previous models (Kalamakis et al., 2019; Ziebell et al., 2018, 2014). The new aspect of the model introduced here is the distinction between resting and dormant NSCs.

The rate r_2 describes the reactivation from the resting state. If r_2 assumes very large values this corresponds to a scenario where the time spent in the resting state before reactivation is negligibly short. In biological terms this means that most cells remain active after division.

We denote the amount of dormant stem cells at time t as $c_d(t)$. The amount of active stem cells at time t is denoted as $c_a(t)$ and that of resting stem cells as $c_r(t)$. For notational convenience we omit the argument t and identify $c_d(t) \equiv c_d$, $c_r(t) \equiv c_r$ and $c_a(t) \equiv c_a$. This results in the following system of ordinary differential equations.

$$
\begin{aligned}
\frac{d}{dt}c_d &= -r_1 c_d && \text{(dormant)}\\
\frac{d}{dt}c_a &= r_2 c_r - c_a p + r_1 c_d && \text{(active)}\\
\frac{d}{dt}c_r &= -r_2 c_r + 2a^2 p c_a + 2(1-a)apc_a && \text{(resting)}\\
&= -r_2 c_r + 2apc_a
\end{aligned}
\tag{1}
$$

$$
\begin{aligned}
c_r(0) &= c_r^0\\
c_a(0) &= c_a^0\\
c_d(0) &= c_d^0
\end{aligned}
\tag{2}
$$

with nonnegative initial conditions c_r^0, c_d^0 and c_a^0.

We note that for $r_1 = r_2$ and $qNSC = c_d + c_r$, where qNSC denotes the amount of quiescent stem cells, we obtain the model from (Kalamakis et al., 2019).

1.4 Model of the labeling experiments

Experimentally we can only count the resting cells that have divided during the EdU exposure and became quiescent afterwards. The obtained cell counts are a lower bound for the total number of resting cells, since resting cells that have not divided during EdU exposure cannot be identified in the experiment. To avoid potential underestimations we explicitly simulate the experimental setup and compare the experimentally obtained resting cell counts to the simulations.

1.4.1 Labeling phase

For the labeling phase we assume:

- During EdU supply all active cells are instantaneously labeled.
- Cells transiting during the time of EdU supply from active to resting state retain the label.

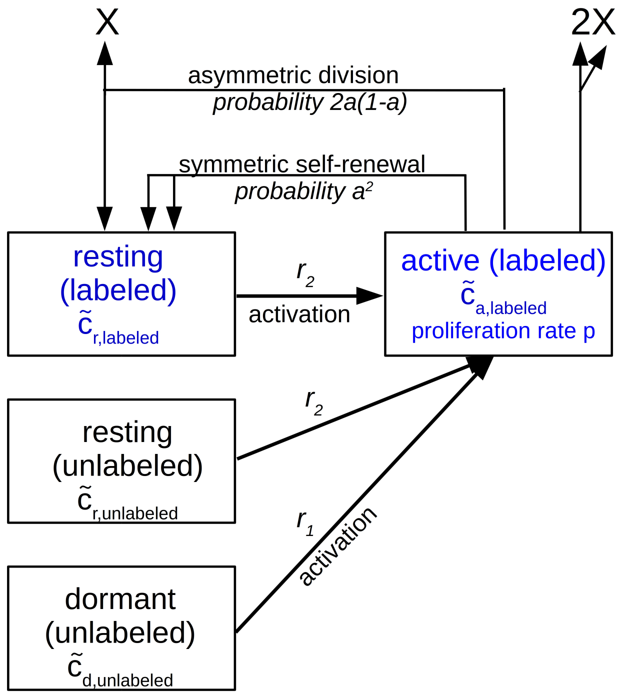

Figure 2: NSC model during EdU supply. The scheme shows processes described by system (3). Non-stem cells are denoted by X.

To quantitatively describe this experiment we have to distinguish between labeled and unlabeled resting cells. Since we assume that active cells get instantaneously labeled all active cells are per definition labeled. During the EdU supply the following model is considered. It is visualized in Figure 2. We denote as $\tilde{c}_d$ the amount of dormant unlabeled cells, as $\tilde{c}_{r,labeled}$ the amount of resting labeled cells, as $\tilde{c}_{r,unlabeled}$ the amount of resting unlabeled cells and as $\tilde{c}_a$ the amount of active labeled cells.

$$
\begin{aligned}
&\frac{d}{dt}\tilde{c}_d = -r_1\tilde{c}_d && \text{(dormant, unlabeled)}\\
&\frac{d}{dt}\tilde{c}_a = r_2(t)(\tilde{c}_{r,labeled} + \tilde{c}_{r,unlabeled}) - p\tilde{c}_a + r_1\tilde{c}_d && \text{(active, labeled)}\\
&\frac{d}{dt}\tilde{c}_{r,labeled} = -r_2(t)\tilde{c}_{r,labeled} + 2a^2p\tilde{c}_a + 2(1-a)ap\tilde{c}_a && \text{(resting, labeled)}\\
&= -r_2(t)\tilde{c}_{r,labeled} + 2ap\tilde{c}_a\\
&\frac{d}{dt}\tilde{c}_{r,unlabeled} = -r_2(t)\tilde{c}_{r,unlabeled} && \text{(resting, unlabeled)}
\end{aligned}
\tag{3}
$$

Assume the EdU supply starts at $t = t^*$, then we have

$$
\begin{aligned}
\tilde{c}_d(t^*) &= c_d(t^*)\\
\tilde{c}_a(t^*) &= c_a(t^*)\\
\tilde{c}_{r,unlabeled}(t^*) &= c_r(t^*)\\
\tilde{c}_{r,labeled}(t^*) &= 0
\end{aligned}
\tag{4}
$$

1.4.2 Chase period

For the chase period we make the following assumptions.

- Unlabeled cells getting activated during the chase period remain unlabeled and produce unlabeled offspring.
- Since the chase period is short and labels are retained during several divisions, we assume that labeled cells dividing during the chase period give rise to labeled cells.

This leads to the following system of equations. We denote as $\hat{c}_{r,labeled}$ the amount of resting labeled cells and as $\tilde{c}_{a,labeled}$ the amount of active labeled cells. The model is visualized in Figure 3.

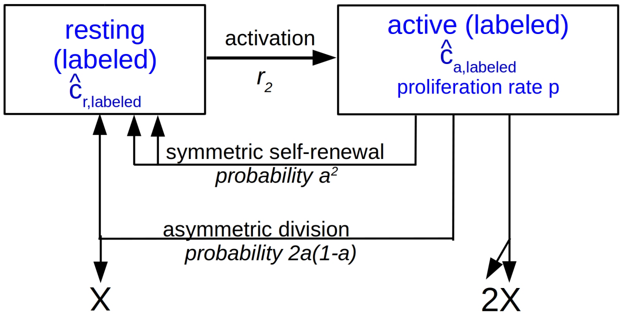

Figure 3: NSC model during the chase period. The scheme shows processes described by system (5). Non-stem cells are denoted by X.

$$\begin{aligned}
\frac{d}{dt}\hat{c}_{a,labeled} &= r_2(t)\hat{c}_{r,labeled} - p\hat{c}_{a,labeled} && \text{(active, labeled)}\\
\frac{d}{dt}\hat{c}_{r,labeled} &= -r_2(t)\hat{c}_{r,labeled} + 2a^2p\hat{c}_{a,labeled} + 2(1-a)ap\hat{c}_{a,labeled} && \text{(resting, labeled)}\\
&= -r_2(t)\hat{c}_{r,labeled} + 2ap\hat{c}_{a,labeled}
\end{aligned} \tag{5}$$

Assume the chase starts at $t = t^{\#}$, then we have

$$\begin{aligned}
&\hat{c}_d(t^{\#}) = \tilde{c}_d(t^{\#})\\
&\hat{c}_{a,labeled}(t^{\#}) = \tilde{c}_a(t^{\#})\\
&\hat{c}_{a,unlabeled}(t^{\#}) = 0\\
&\hat{c}_{r,unlabeled}(t^{\#}) = \tilde{c}_{r,unlabeled}(t^{\#})\\
&\hat{c}_{r,labeled}(t^{\#}) = \tilde{c}_{r,labeled}(t^{\#})
\end{aligned} \tag{6}$$

1.5 Full model

The amount of resting (EdU+Ki67-) cells present at time t is obtained as follows. Time evolution of active, resting and dormant NSCs before EdU supply is simulated using system (1) with initial conditions (2). We start simulation at time 0 and stop at $t^* = t$. Then we

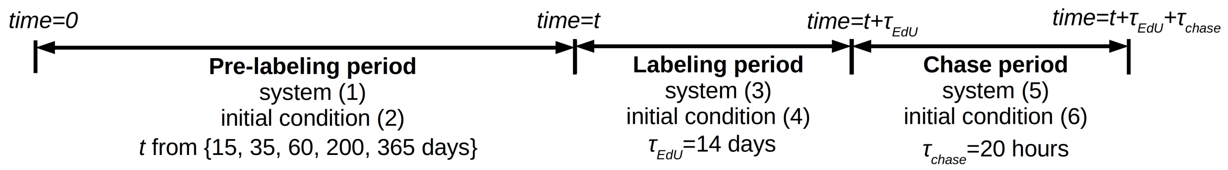

Figure 4: Simulation of labeling experiments.

simulate the labeling period of $\tau_{EdU} = 14$ days using system (3) with the initial condition (4) for $t^* = t$. We then simulate the chase using system (5) with the initial condition (6) for $t^{\#} = t^* + \tau_{EdU}$. We stop simulations at $t = t^* + \tau_{EdU} + \tau_{chase}$, with $\tau_{chase} = 20$ hours to readout the resting labeled cell count to compare it to measurements. The procedure is summarized in Figure 4.

2 Model simulations

The model has been implemented in MATLAB and ordinary differential equations have been solved using the solver *ode23s*.

3 Model quantification

We assume a doubling time of active NSCs of 22.8 hours, as measured in (Brandt et al., 2012). The other model parameters and the initial conditions c_a^0, c_r^0, c_d^0 are fitted based on the data. For fitting we use a multistart approach (5000 multistarts) with random nonnegative initial parameter guesses. The sampling of the initial guesses follows a latin hypercube approach. Optimization is performed using the MATLAB function *fmincon*.

4 Model selection

To compare different models we use the Akaike information criterion for small sample sizes (AIC_c) given as

$$AIC_c = AIC + \frac{2k^2 + 2k}{n - k - 1},$$

where n is the number of data points, k the number of free parameters and AIC the Akaike information criterion (Burnham and Anderson, 2002). This approach takes into account

the accuracy of the fit but at the same time punishes a high number of free parameters to prevent overfitting. The level of empirical support of a given model is considered substantial if $0 \le \Delta \le 2$, considerably less so if $4 \le \Delta \le 7$ and none, if $\Delta > 10$ holds, where Δ is the AIC_c of a given model minus the minimum AIC_c of all considered models (Burnham and Anderson, 2002).

5 Age-related change of cell parameters

In (Kalamakis et al., 2019; Ziebell et al., 2018) it has been concluded that the activation rate of NSCs changes during aging. This can be modeled by assuming that the rate is not given by a constant but by a time-dependent function. In the following we allow the activation rates r_1, r_2 and the self-renewal probability a to be functions of the age of the organism.

6 Fitting active and total NSC dynamics

To check consistency of our dataset with the model from (Kalamakis et al., 2019) we first fit different versions of the model only to active and total NSC counts, i.e., for the moment we ignore the information about resting NSCs.

6.1 Identical activation rates for dormant and resting NSCs

In (Kalamakis et al., 2019; Ziebell et al., 2018) it has been concluded that the activation rate of quiescent NSCs changes during aging. To obtain the model from (Kalamakis et al., 2019) we assume that the activation rates are identical for dormant and resting cells and that they change with age. As in (Kalamakis et al., 2019) we set

$$r(t) := r_1(t) = r_2(t) = r_{max}e^{-\beta_r t}.$$

We assume a cell cycle time of active NSCs of 22.8 hours, as measured in (Brandt et al., 2012). We fit the other model parameters to active and total NSC counts. We observe that the model is close to the data, however, the values of the estimated parameters differ from those in (Kalamakis et al., 2019).

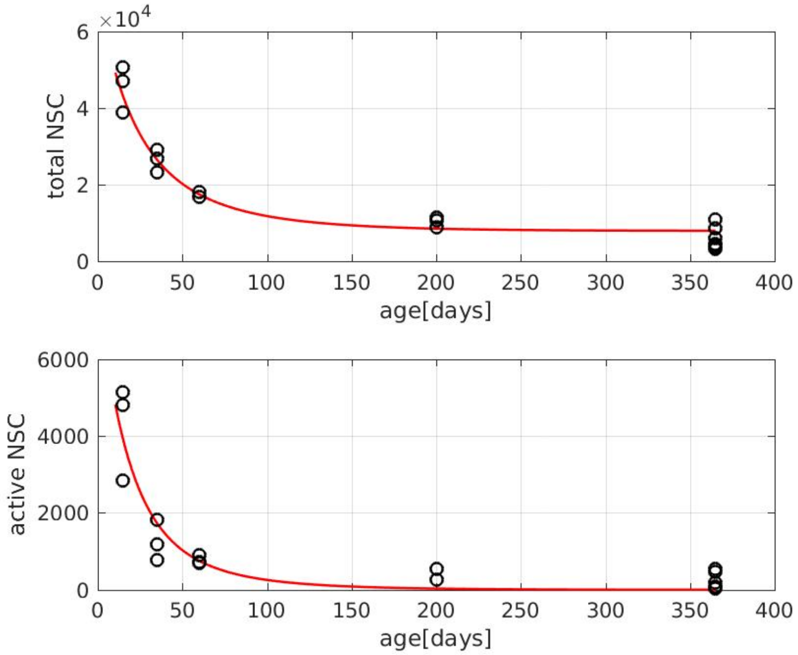

Parameter	Value	Source	Parameter	Value	Source
r_{max} [1/days]	0.1304	fitted	β_r [1/days]	0.0174	fitted
a	0.3518	fitted	p [1/days]	1.0526	(Brandt et al., 2012)

AIC_c=33.1

6.2 Different activation rates for dormant and resting NSCs

We now consider the case where activation rates of dormant and resting NSCs can be different. As in (Ziebell et al., 2018; Kalamakis et al., 2019) we assume that they decline exponentially with time, i.e.,

$$r_1(t) = r_{max,1}e^{-\beta_{r,1}t},$$
$$r_2(t) = r_{max,2}e^{-\beta_{r,2}t}.$$

We fit this version of the model to the counts of total and active NSCs. This does not improve the fit and an increase in AIC_c demonstrates that based on the total and active cell counts there is no evidence for the rates to be different.

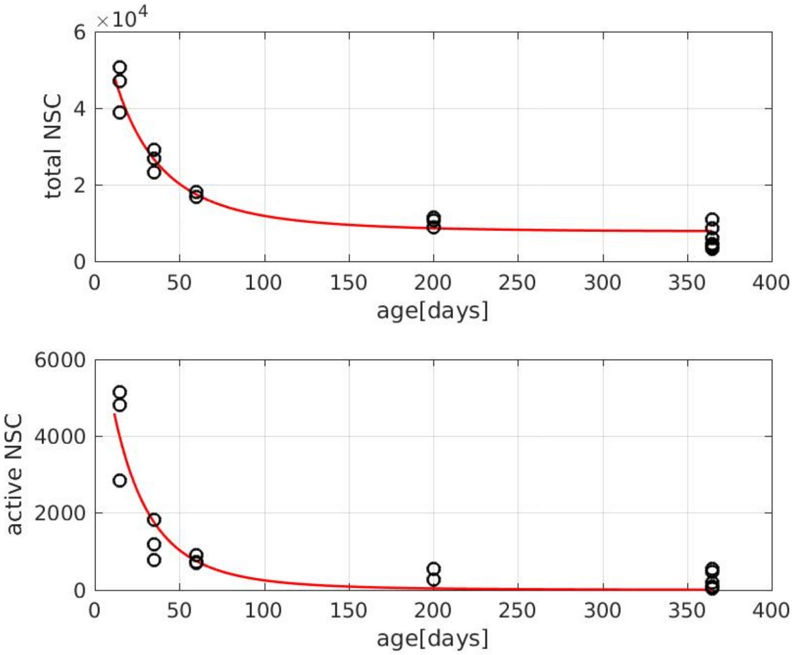

Parameter	Value	Source	Parameter	Value	Source
$r_{max,1}$ [1/days]	0.8211	fitted	$\beta_{r,1}$ [1/days]	0.9924	fitted
$r_{max,2}$ [1/days]	0.1404	fitted	$\beta_{r,2}$ [1/days]	0.0111	fitted
a	0.3508	fitted	p [1/days]	1.0526	(Brandt et al., 2012)

AIC_c=38.3

7 Fitting total, active and resting NSC dynamics

Now we include our data on resting NSCs. In this section we fit the model to total, active and resting NSC counts.

7.1 Identical activation rate for dormant and resting NSCs

We first consider the scenario where dormant and resting NSCs have the same activation rate, i.e., we set

$$r(t) := r_1(t) = r_2(t) = r_{max}e^{-\beta_r t}.$$

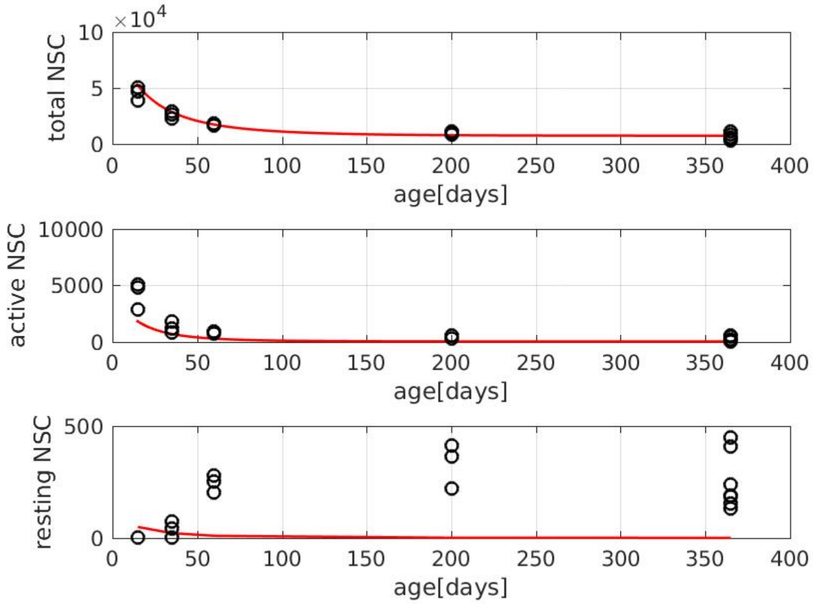

Parameter	Value	Source	Parameter	Value	Source
r_{max} [1/days]	0.0467	fitted	β_r [1/days]	0.0185	fitted
a	0.0016	fitted	p [1/days]	1.0526	(Brandt et al., 2012)

AIC_c=120.3

7.2 Different activation rates for dormant and resting NSCs

If we allow dormant and resting cells to have different activation rates, i.e.,

$$r_1(t) = r_{1,max}e^{-\beta_{r1}t},$$

$$r_2(t) = r_{2,max}e^{-\beta_{r2}t},$$

we obtain a better fit and a reduction in AIC_c. This suggests that different activation rates are required to explain the observed dynamics of resting NSCs.

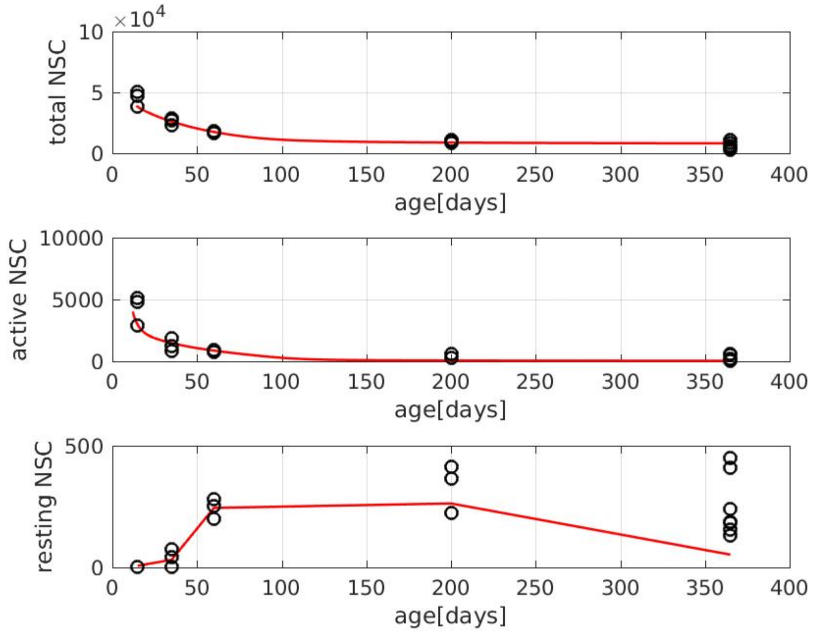

Parameter	Value	Source	Parameter	Value	Source
$r_{max,1}$ [1/days]	0.0202	fitted	$\beta_{r,1}$ [1/days]	0.0053	fitted
$r_{max,2}$ [1/days]	3390.0	fitted	$\beta_{r,2}$ [1/days]	0.1028	fitted
a	0.3536	fitted	p [1/days]	1.0526	(Brandt et al., 2012)

AIC_c=53.3

The high activation rate of resting cells indicates that in young mice most activated cells do not return to quiescence after division.

7.3 Different activation rates for dormant and resting NSCs and age dependent self-renewal

We now allow different activation rates for dormant and resting cells and we allow self-renewal to increase over time. The expression for age-dependent self-renewal has been taken from (Kalamakis et al., 2019). We set

$$r_1(t) = r_{1,max}e^{-\beta_{r1}t},$$

$$r_2(t) = r_{2,max}e^{-\beta_{r2}t},$$

$$a = \frac{1}{2} \cdot (1 + e^{-\beta_a t} \cdot (2a_{min} - 1)).$$

This best obtained fit is the following.

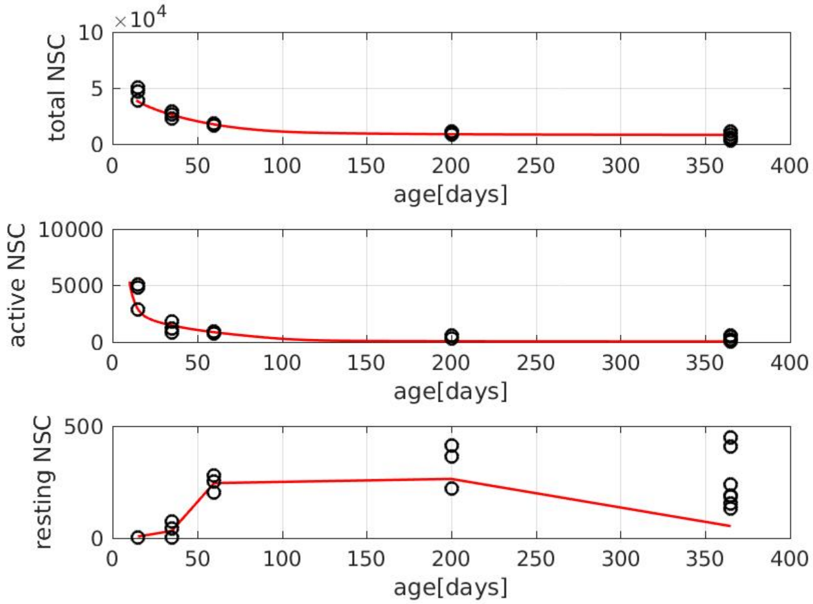

Parameter	Value	Source	Parameter	Value	Source
$r_{max,1}$ [1/days]	0.0202	fitted	$\beta_{r,1}$ [1/days]	0.0053	fitted
$r_{max,2}$ [1/days]	3389.3	fitted	$\beta_{r,2}$ [1/days]	0.1029	fitted
a_{min}	0.3526	fitted	β_a [1/days]	$7.25 \cdot 10^{-5}$	fitted
p [1/days]	1.0526	(Brandt et al., 2012)			

AIC_c=56.1

We observe an increase in AIC_c supporting the view that age dependence of self-renewal has only a minor impact on cell dynamics. Also here the high activation rate of resting cells indicates that in young mice most activated cells do not return to quiescence after division.

The following figure shows how activation rates and self-renewal probability change with age.

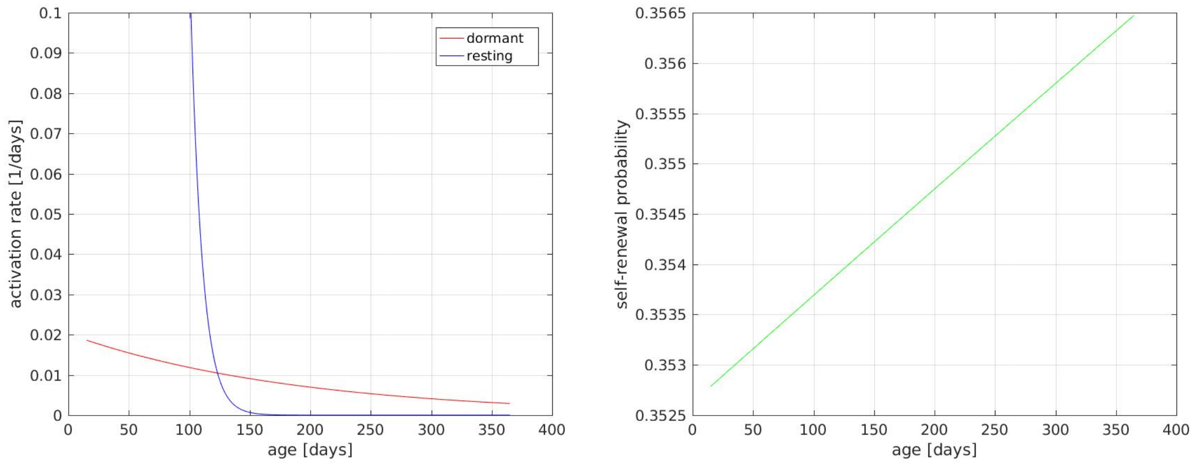

7.4 Model with non-zero activation for large time

In the versions of the model considered so far, the activation rates decline asymptotically with time. This can lead to rates that are practically zero within the life-time of a mouse. To prevent this unrealistic scenario, we modified the model as follows:

$$r_1 = r_{1,max}e^{-\beta_{r1}t} + \epsilon,$$

$$r_2 = r_{2,max}e^{-\beta_{r2}t} + \epsilon.$$

This implies that the activation rate is always larger than ϵ. We fit ϵ in addition to the other parameters.

7.4.1 Constant self-renewal

We first consider the scenario with constant in time self-renewal ($a = const$) and age-dependent activation rates:

$$r_1 = r_{1,max}e^{-\beta_{r1}t} + \epsilon,$$

$$r_2 = r_{2,max}e^{-\beta_{r2}t} + \epsilon.$$

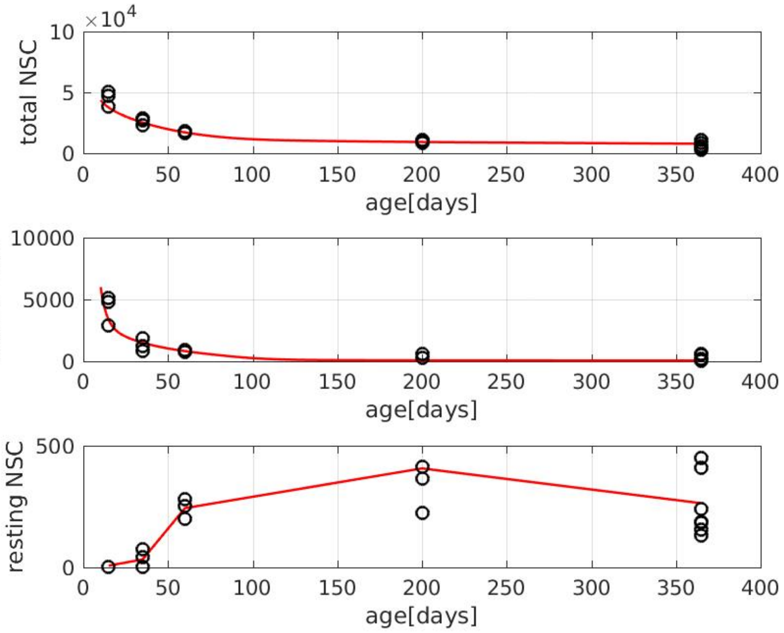

Parameter	Value	Source	Parameter	Value	Source
$r_{max,1}$ [1/days]	0.0196	fitted	$\beta_{r,1}$ [1/days]	0.0107	fitted
$r_{max,2}$ [1/days]	3390.0	fitted	$\beta_{r,2}$ [1/days]	0.1035	fitted
a	0.3604	fitted	ϵ [1/days]	0.0031	fitted
p [1/days]	1.0526	(Brandt et al., 2012)			

AIC_c=36.0

The fit is significantly improved. Although we have one more free parameter AIC_c is reduced compared to the previous versions of the model. This implies that it is important for the observed process that activation rates do not decline to zero for high ages.

7.4.2 Age-dependent self-renewal

We now allow in addition that self-renewal is age-dependent. As motivated above, we set

$$r_1 = r_{1,max} e^{-\beta_{r1} t} + \epsilon,$$

$$r_2 = r_{2,max} e^{-\beta_{r2} t} + \epsilon,$$

$$a = \frac{1}{2} \cdot (1 + e^{-\beta_a t} \cdot (2a_{min} - 1)).$$

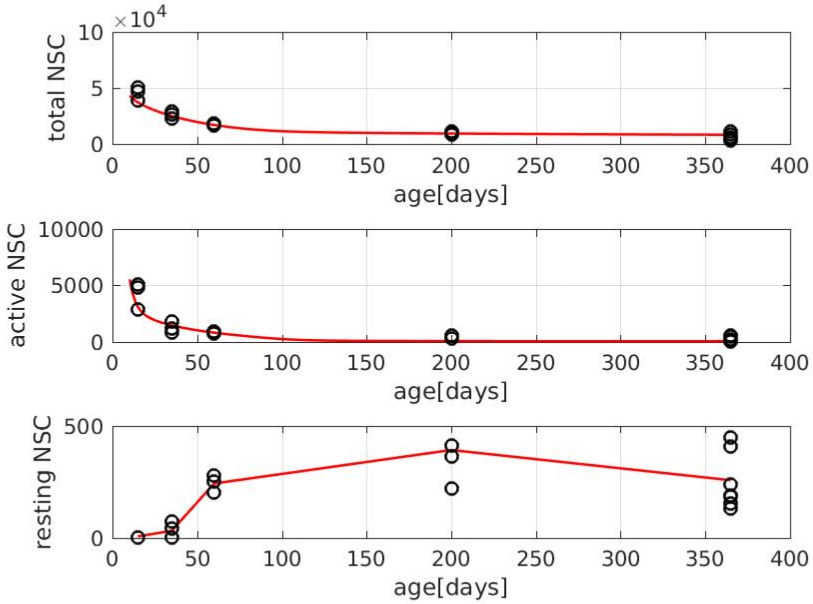

Parameter	Value	Source	Parameter	Value	Source
$r_{max,1}$ [1/days]	0.0202	fitted	$\beta_{r,1}$ [1/days]	0.0102	fitted
$r_{max,2}$ [1/days]	3390.0	fitted	$\beta_{r,2}$ [1/days]	0.1033	fitted
a_{min}	0.3537	fitted	β_a [1/days]	$8.1 \cdot 10^{-4}$	fitted
ϵ [1/days]	0.0026	fitted	p [1/days]	1.0526	(Brandt et al., 2012)

AIC_c=41.4

The increase in AIC_c indicates that age-dependence of self-renewal has only little impact on the NSC dynamics.

The following figure shows how activation rates and self-renewal probability change with age.

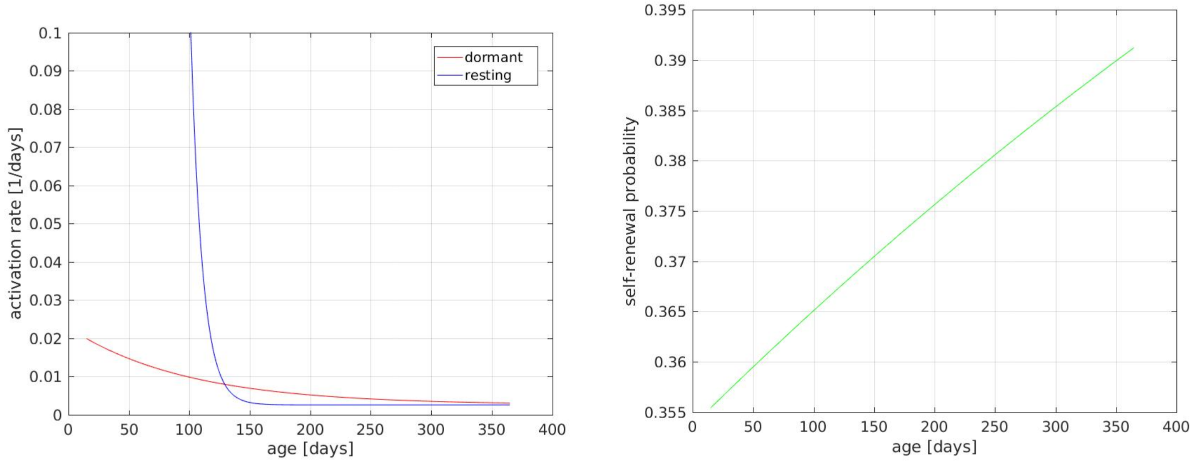

7.5 Age-dependent proliferation

Compared to the model version in section 7.4.1 we now additionally consider age-dependent proliferation. Therefore, we set

$$r_1 = r_{1,max}e^{-\beta_{r1}t} + \epsilon,$$
$$r_2 = r_{2,max}e^{-\beta_{r2}t} + \epsilon,$$
$$p = p_{max}e^{-\beta_{p}t}.$$

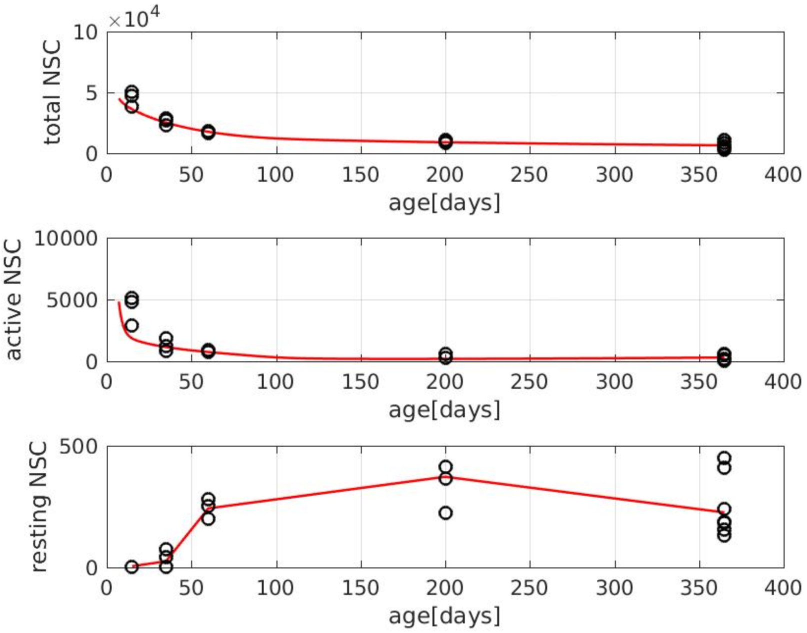

Parameter	Value	Source	Parameter	Value	Source
$r_{max,1}$ [1/days]	0.0182	fitted	$\beta_{r,1}$ [1/days]	0.0134	fitted
$r_{max,2}$ [1/days]	3390.7	fitted	$\beta_{r,2}$ [1/days]	0.1132	fitted
a	0.2855	fitted	p_{max} [1/days]	1.0526	(Brandt et al., 2012)
β_p [1/days]	0.0064	fitted	ϵ[1/days]	0.0045	fitted

AIC_c=37.9

The AIC_c reveals that this model is not more feasible compared to the model in section 7.4.1. The parametrization in this section would imply a cell cycle duration of approx. 3 days in mice of 6 months of age, which is longer than measured in experiments (Brandt et al., 2012). When we consider p_{max} as an additional free parameter and fit it based on the data, the AIC_c slightly decreases ($AIC_c = 35.0$), however in this case NSC cell cycle duration at 6 months of age would be approx. 2.7 days, which is longer than inferred from experimental measurements (Brandt et al., 2012). We, therefore, stick to the model from section 7.4.1.

7.6 Age-dependent self-renewal and proliferation

Compared to the model version in section 7.4.1 we now additionally consider age-dependent proliferation and self-renewal. Therefore, we set

$$r_1 = r_{1,max}e^{-\beta_{r1}t} + \epsilon,$$
$$r_2 = r_{2,max}e^{-\beta_{r2}t} + \epsilon,$$
$$a = \frac{1}{2} \cdot (1 + e^{-\beta_a t} \cdot (2a_{min} - 1)),$$
$$p = p_{max}e^{-\beta_p t}.$$

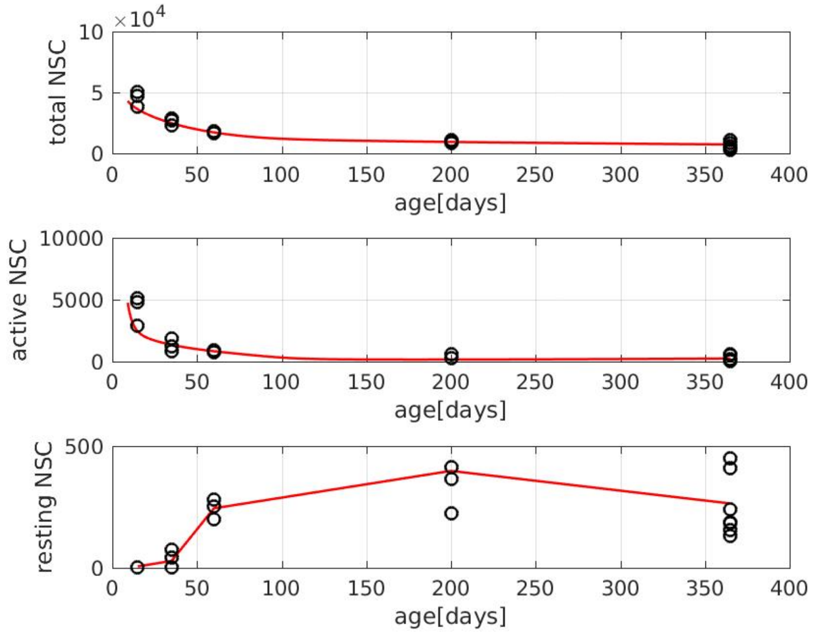

Parameter	Value	Source	Parameter	Value	Source
$r_{max,1}$ [1/days]	0.0200	fitted	$\beta_{r,1}$ [1/days]	0.0142	fitted
$r_{max,2}$ [1/days]	3388.7	fitted	$\beta_{r,2}$ [1/days]	0.1095	fitted
a_{min}	0.3211	fitted	β_a [1/days]	$9.38 \cdot 10^{-7}$	fitted
ϵ [1/days]	0.0041	fitted	p_{max} [1/days]	1.0526	(Brandt et al., 2012)
β_p [1/days]	0.0056	fitted			

AIC_c=36.3

The AIC_c is similar to the AIC_c of the model from section 7.4.1 implying that the age-dependent variability of the proliferation rate does not improve the model.

The parametrization in this section would imply a NSC cell cycle duration of approx. 2.6 days in mice of 6 months of age, which is longer than measured in experiments

(Brandt et al., 2012). When we consider p_{max} as an additional free parameter and fit it based on the data, the model can be improved ($AIC_c = 28.2$), however in this case NSC cell cycle duration at 6 months of age would be approx. 3.7 days, which is unrealistic. We, therefore, stick to the model from section 7.4.1.

8 Study of specific scenarios

In this section, two alternate scenarios are explored. Firstly, a model where proliferation rate is the only age-dependent parameter. And a second scenario where there is no self-renewal. We consider these models to be more extreme scenarios as they are contradicted by previous experimental observations (Ziebell et al., 2018; Kalamakis et al., 2019; Encinas et al., 2011; Pilz et al., 2018; Bonaguidi et al., 2011). The results in this section imply that age-dependent activation rates are crucial to capture the experimentally observed NSC dynamics.

8.1 Proliferation rate as only age-dependent cell parameter

To study model dynamics under the assumption that only proliferation rate is age-dependent, we set $\beta_{r,1} = 0$, $\beta_{r,2} = 0$, $\beta_a = 0$. In this case model dynamics do not agree with experiments.

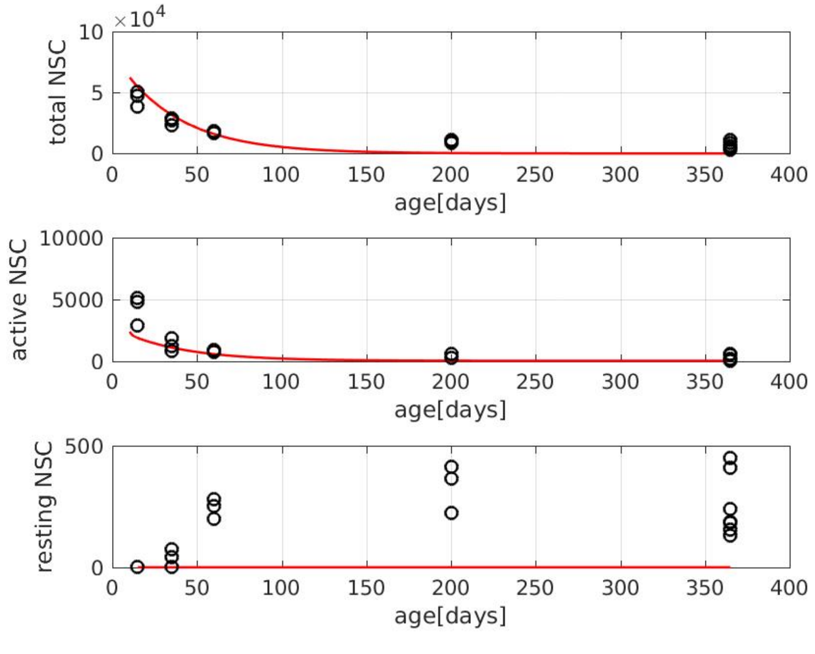

Parameter	Value	Source	Parameter	Value	Source
$r_{max,1}$ [1/days]	0.0263	fitted	$r_{max,2}$ [1/days]	8139.5	fitted
a [1/days]	0.2995	fitted	p [1/days]	2.0166	fitted
β_p [1/days]	0.000307	fitted	ϵ [1/days]	0.0010	fitted

AIC_c=152.0

8.2 Scenarios without self-renewal

To understand the impact of self-renewal on NSC dynamics we consider two scenarios. First, we use the fitted model from section 7.4.1 and set self-renewal to zero. This allows to quantify the impact of self-renewal on NSC population dynamics in the model. Naturally, in absence of self-renewal there exist no resting NSCs, since offspring of NSCs belong to more differentiated cell types. Furthermore, the total cell dynamics cannot be recovered by the model, since in absence of self-renewal the number of active NSCs in young mice is smaller than observed in experiments.

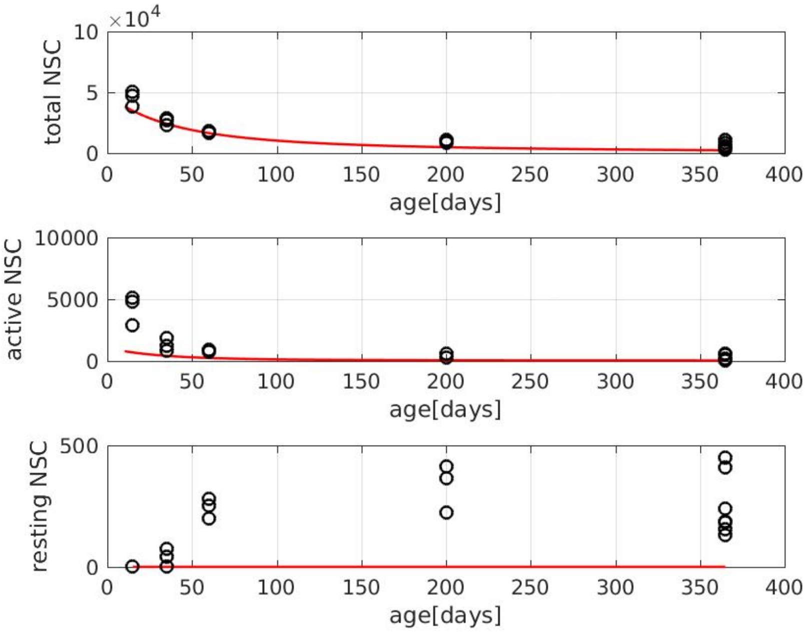

As second approach we re-estimated the cell parameters subject to the constraint that self-renewal equals zero. Also in this case the ratio of active and quiescent cells is not captured by the model. This agrees with the observations in (Kalamakis et al., 2019; Ziebell et al., 2018).

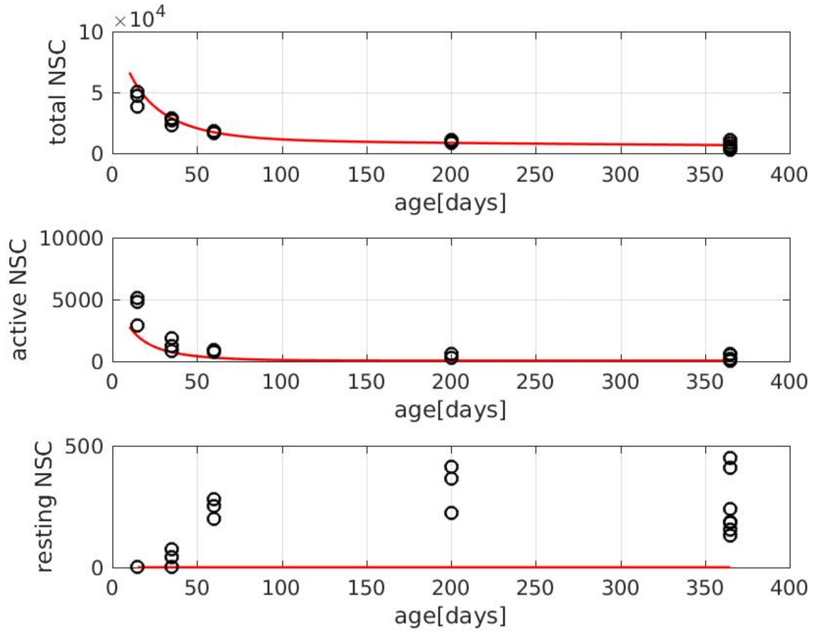

Parameter	Value	Source	Parameter	Value	Source
$r_{max,1}$ [1/days]	0.0333	fitted	$\beta_{r,1}$ [1/days]	$2.625{\cdot}10^{-09}$	fitted
$r_{max,2}$ [1/days]	0.0692	fitted	$\beta_{r,2}$ [1/days]	0.0388	fitted
a_{min}	0	fixed	p [1/days]	1.0526	(Brandt et al., 2012)
ϵ[1/days]	0.0014	fitted			

AIC_c=125.3

Based on these simulations we conclude that NSC self-renewal is required to recapitulate experimentally observed NSC population dynamics.

9 Model with different EdU administration periods

Active NSCs persist for longer with age (Figure 1L, main text). Therefore, it might be important to lengthen the EdU administration period in older mice so as to not underestimate the number of resting NSCs. A subset of 12-month old mice (n = 5) received a 28-day EdU exposure to test whether lengthening the administration period would affect counts of resting NSCs.

These different EdU administration periods can be taken into account in the model, however it leads only to slight changes of the estimated parameters. As an example, we

consider in this section an extended version of the model from section 7.4.1 (model with age-dependent activation and age-independent proliferation and self-renewal) that considers the different EdU administration periods.

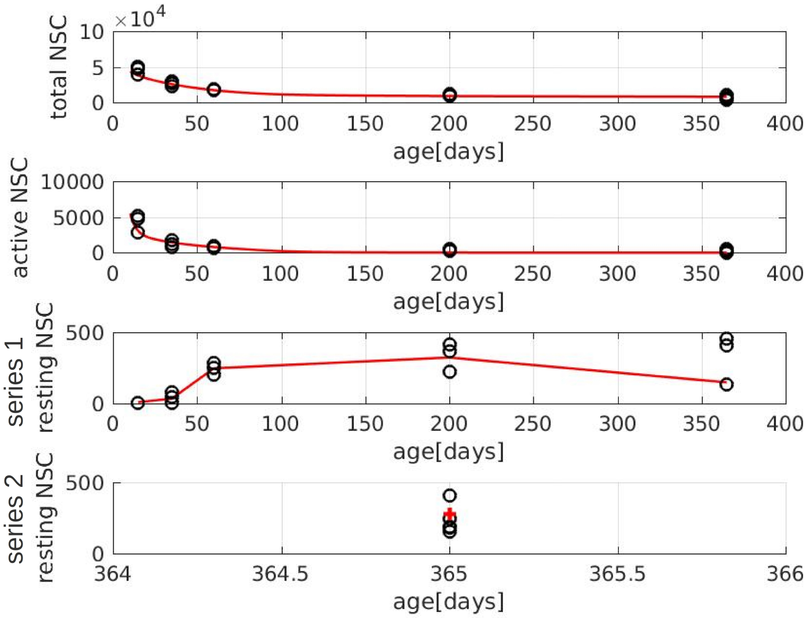

The series of experiments with EdU administration periods of 14 days is referred to as 'series 1' (third row). The series of experiments with EdU administration periods of 28 days is referred to as 'series 2' (fourth row). In the fourth row the model solution is shown as a red cross (+).

Parameter	Value	Source	Parameter	Value	Source
$r_{max,1}$ [1/days]	0.02	fitted	$\beta_{r,1}$ [1/days]	0.0074	fitted
$r_{max,2}$ [1/days]	3396.9	fitted	$\beta_{r,2}$ [1/days]	0.1034	fitted
a	0.3557	fitted	ϵ [1/days]	0.0014	fitted
p [1/days]	1.0526	(Brandt et al., 2012)			

$AIC_c = 43.8^\star$

$\star$ This AIC_c cannot be directly compared to the AIC_c values of the previous model fits, since here the model has been fitted to a different dataset taking into account different EdU administration periods.

We note that the number of experimentally measured resting cells does not differ significantly for experiments with 14 days EdU administration compared to experiments with 28

days EdU administration. For this reason we consider the simpler model from section 7.4.1 as a suitable approximation of the experiments.

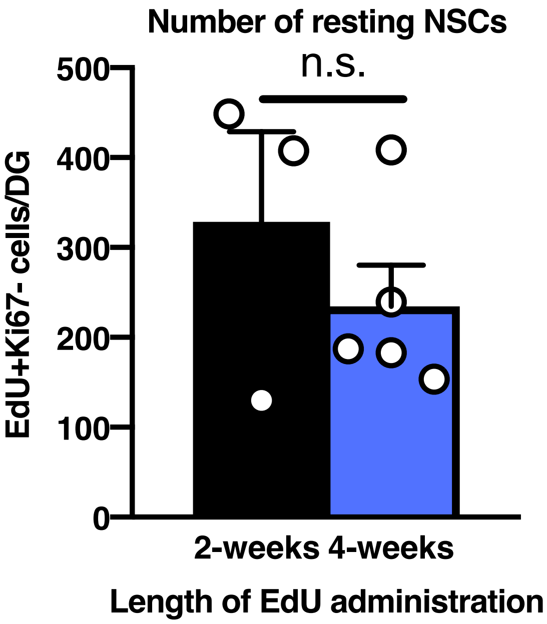

The number of EdU+Ki67- cells per dentate gyrus does not differ in experiments with 14 days EdU administration (left) compared to experiments with 28 days EdU administration (right). Significance level 0.05, two-sided t-test.

10 Model without return to quiescence

Our MCM2 immunolabeling studies (Figure S1) show active cells return to a quiescent (resting) state. However, in this section we test a hypothetical scenario where NSCs do not return to quiescence and instead reside in an extended G1 phase. These hypothetical G1 phases have no upper time-limits.

Dormant cells are activated at rate r_1. Activated NSCs divide at rate p. After each NSC division the fraction a of the offspring are NSCs and the fraction $1 - a$ adopt a more differentiated phenotype. Cells originating from division enter G1 phase, from which they are reactivated at rate r_2. We furthermore assume that during its nth division a NSC only gives rise to more differentiated cells. Fig. 5 provides an overview of the model structure.

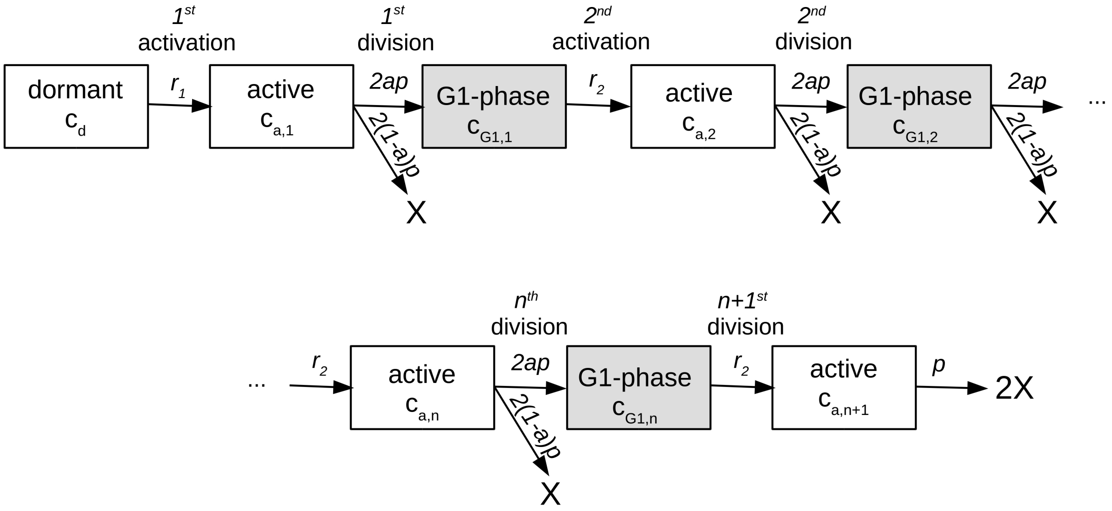

Figure 5: Model without return to quiescence. Non-stem cells are denoted by X.

Denote by $c_d(t) \equiv c_d$ the amount of dormant NSCs at time t, by $c_{a,i}$ the amount of NSCs that have been activated i times $(1 \le i \le n+1)$ since their exit from dormancy and by $c_{G1,i}$ the amount of NSCs that are in the G1 phase for the ith time since their exit from dormancy. This results in the following system of ordinary differential equations:

$$
\begin{aligned}
\frac{d}{dt}c_d &= -r_1 c_d && \text{(dormant)}\\
\frac{d}{dt}c_{a,1} &= r_1 c_d - p c_{a,1} && \text{(activated for the } 1^{st} \text{ time)}\\
\frac{d}{dt}c_{G1,1} &= 2ap c_{a,1} - r_2 c_{G1,1} && \text{(G1 for the } 1^{st} \text{ time)}\\
\frac{d}{dt}c_{a,2} &= r_2 c_{G1,1} - p c_{a,2} && \text{(activated for the } 2^{nd} \text{ time)}\\
\frac{d}{dt}c_{G1,2} &= 2ap c_{a,2} - r_2 c_{G1,2} && \text{(G1 for the } 2^{nd} \text{ time)}\\
\dots & && \dots\\
\frac{d}{dt}c_{a,n} &= r_2 c_{G1,n-1} - p c_{a,n} && \text{(activated for the } n^{th} \text{ time)}\\
\frac{d}{dt}c_{G1,n} &= 2ap c_{a,n} - r_2 c_{G1,n} && \text{(G1 for the } n^{th} \text{ time)}\\
\frac{d}{dt}c_{a,n+1} &= r_2 c_{G1,n} - p c_{a,n+1} && \text{(activated for the } n+1^{st} \text{ time)},
\end{aligned}
\tag{7}
$$

supplemented by the following initial conditions:

$$
\begin{aligned}
c_d(0) &= c_d^0\\
c_{a,i}(0) &= c_{a,i}^0, 1 \le i \le n+1\\
c_{G1,i}(0) &= c_{G1,i}^0, 1 \le i \le n.
\end{aligned}
$$

As above we set

$$
\begin{aligned}
r_1 &= r_{1,max} e^{-\beta_{r1} t} + \epsilon,\\
r_2 &= r_{2,max} e^{-\beta_{r2} t} + \epsilon.
\end{aligned}
$$

10.1 Labeling phase

We make the same assumptions as in section 1.4.1, i.e., active cells get immediately labeled when EdU supply starts. We denote by $\tilde{c}_d$ the amount of dormant NSCs at time t, by $\tilde{c}_{a,i}$ the amount of labeled NSCs that have been activated i times ($1 \le i \le n+1$) since their exit from dormancy, by $c_{G1,i,l}$ the amount of labeled NSCs that are in the G1 phase for the ith time since their exit from dormancy and by $c_{G1,i,u}$ the amount of unlabeled NSCs that are in

the G1 phase for the ith time since their exit from dormancy. This results in the following system of equations:

$$
\begin{aligned}
\frac{d}{dt}\tilde{c}_d &= -r_1\tilde{c}_d && \text{(dormant, unlabeled)}\\
\frac{d}{dt}\tilde{c}_{a,1} &= r_1\tilde{c}_d - p\tilde{c}_{a,1} && \text{(activated for the } 1^{st} \text{ time, labeled)}\\
\frac{d}{dt}\tilde{c}_{G1,1,l} &= 2ap\tilde{c}_{a,1} - r_2\tilde{c}_{G1,1,l} && \text{(G1 for the } 1^{st} \text{ time, labeled)}\\
\frac{d}{dt}\tilde{c}_{G1,1,u} &= -r_2\tilde{c}_{G1,1,u} && \text{(G1 for the } 1^{st} \text{ time, unlabeled)}\\
\frac{d}{dt}\tilde{c}_{a,2} &= r_2(\tilde{c}_{G1,1,l} + \tilde{c}_{G1,1,u}) - p\tilde{c}_{a,2} && \text{(activated for the } 2^{nd} \text{ time, labeled)}\\
\frac{d}{dt}\tilde{c}_{G1,2,l} &= 2ap\tilde{c}_{a,2} - r_2\tilde{c}_{G1,2,l} && \text{(G1 for the } 2^{nd} \text{ time, labeled)}\\
\frac{d}{dt}\tilde{c}_{G1,2,u} &= -r_2\tilde{c}_{G1,2,u} && \text{(G1 for the } 2^{nd} \text{ time, unlabeled)}\\
&\cdots && \cdots\\
\frac{d}{dt}\tilde{c}_{a,n} &= r_2(\tilde{c}_{G1,n-1,l} + \tilde{c}_{G1,n-1,u}) - p\tilde{c}_{a,n} && \text{(activated for the } n^{th} \text{ time, labeled)}\\
\frac{d}{dt}\tilde{c}_{G1,n,l} &= 2ap\tilde{c}_{a,n} - r_2\tilde{c}_{G1,n,l} && \text{(G1 for the } n^{th} \text{ time, labeled)}\\
\frac{d}{dt}\tilde{c}_{G1,n,u} &= -r_2\tilde{c}_{G1,n,u} && \text{(G1 for the } n^{th} \text{ time, unlabeled)}\\
\frac{d}{dt}\tilde{c}_{a,n+1} &= r_2(\tilde{c}_{G1,n,l} + \tilde{c}_{G1,n,u}) - p\tilde{c}_{a,n+1} && \text{(activated for the } n+1^{st} \text{ time, labeled)}
\end{aligned}
\tag{8}
$$

Assume the EdU supply starts at $t = t^*$, then we have

$$
\begin{aligned}
&\tilde{c}_d(t^*) = c_d(t^*)\\
&\tilde{c}_{a,i}(t^*) = c_{a,i}(t^*),\ i = 1,\ ...,\ n+1\\
&\tilde{c}_{G1,i,u}(t^*) = c_{G1,i}(t^*),\ i = 1,\ ...,\ n\\
&\tilde{c}_{G1,i,l}(t^*) = 0,\ i = 1,\ ...,\ n.
\end{aligned}
$$

10.2 Chase period

We make the same assumptions as in section 1.4.2. We denote $\hat{c}_{a,i}$ the amount of labeled NSCs that have been activated i times ($1 \leq i \leq n+1$) since their exit from dormancy and

by $c_{G1,i,l}$ the amount of labeled NSCs that are in the G1 phase for the ith time since their exit from dormancy. The following system of equations describes the evolution of labeled NSCs during the chase period.

$$
\begin{aligned}
\frac{d}{dt}\hat{c}_{a,1} &= -p\hat{c}_{a,1} && \text{(activated for the } 1^{st} \text{ time, labeled)}\\
\frac{d}{dt}\hat{c}_{G1,1,l} &= 2ap\hat{c}_{a,1} - r_2\hat{c}_{G1,1,l} && \text{(G1 for the } 1^{st} \text{ time, labeled)}\\
\frac{d}{dt}\hat{c}_{a,2} &= r_2\hat{c}_{G1,1,l} - p\hat{c}_{a,2} && \text{(activated for the } 2^{nd} \text{ time, labeled)}\\
\frac{d}{dt}\hat{c}_{G1,2,l} &= 2ap\hat{c}_{a,2} - r_2\hat{c}_{G1,2,l} && \text{(G1 for the } 2^{nd} \text{ time, labeled)}\\
&\dots && \dots\\
\frac{d}{dt}\hat{c}_{a,n} &= r_2\hat{c}_{G1,n-1,l} - p\hat{c}_{a,n} && \text{(activated for the } n^{th} \text{ time, labeled)}\\
\frac{d}{dt}\hat{c}_{G1,n,l} &= 2ap\hat{c}_{a,n} - r_2\hat{c}_{G1,n,l} && \text{(G1 for the } n^{th} \text{ time, labeled)}\\
\frac{d}{dt}\hat{c}_{a,n+1} &= r_2\hat{c}_{G1,n,l} - p\hat{c}_{a,n+1} && \text{(activated for the } n+1^{st} \text{ time, labeled)}
\end{aligned}
\tag{9}
$$

Assume the chase starts at $t = t^{\#}$, then we have the following initial conditions

$$
\begin{aligned}
\hat{c}_{a,i}(t^{\#}) &= \tilde{c}_{a,i}(t^{\#}),\ i = 1,\ ...,\ n+1\\
\hat{c}_{G1,i,l}(t^{\#}) &= \tilde{c}_{G1,i,l}(t^{\#}),\ i = 1,\ ...,\ n.
\end{aligned}
$$

10.3 Fitting

We assume a doubling time of active NSCs of 22.8 hours, as measured in (Brandt et al., 2012). The other model parameters are fitted based on the data. For the initial condition we assume a quasi steady state, namely $c^0_{a,1} = \frac{r_1 c^0_d}{p}$, $c^0_{G1,1} = \frac{2apc^0_{a,1}}{r_2}$, $c^0_{a,i} = \frac{r_2 c_{G1,i-1}}{p}$ $(2 \le i \le n+1)$, $c^0_{G1,i} = \frac{2apc^0_{a,i}}{r_2}$ $(2 \le i \le n)$. We assume that dormant NSCs and NSCs in the G1 phase are Ki67-. For fitting we use a multistart approach (5000 multistarts) with random nonnegative initial parameter guesses. The sampling of the initial guesses follows a latin hypercube approach. Optimization is performed using the MATLAB function *fmincon*.

We performed fits for different numbers of n.

10.3.1 Maximal number of 5 divisions

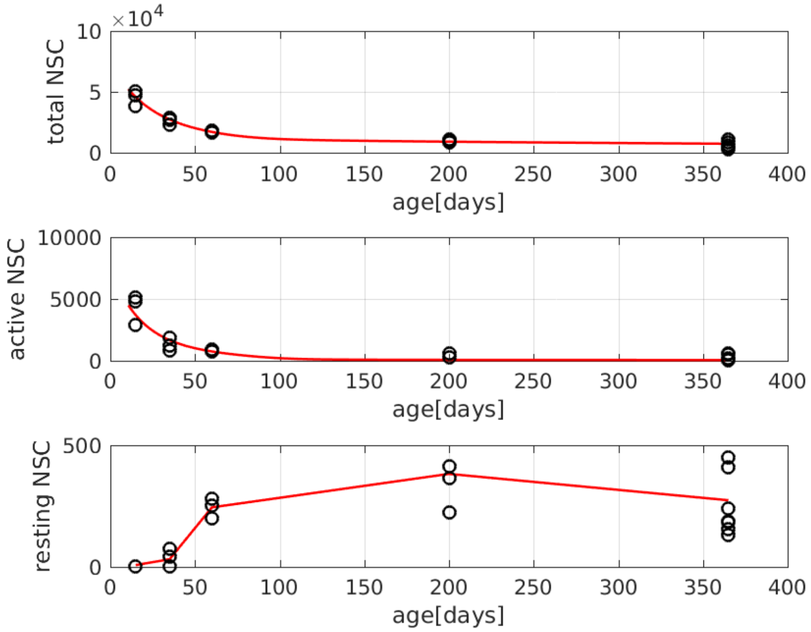

Parameter	Value	Source	Parameter	Value	Source
$r_{max,1}$ [1/days]	0.0318	fitted	$\beta_{r,1}$ [1/days]	0.0107	fitted
$r_{max,2}$ [1/days]	3692.0	fitted	$\beta_{r,2}$ [1/days]	0.1084	fitted
a	0.3471	fitted	ϵ [1/days]	0.0037	fitted
p [1/days]	1.0526	(Brandt et al., 2012)			

AIC_c=25.1

10.3.2 Maximal number of 10 divisions

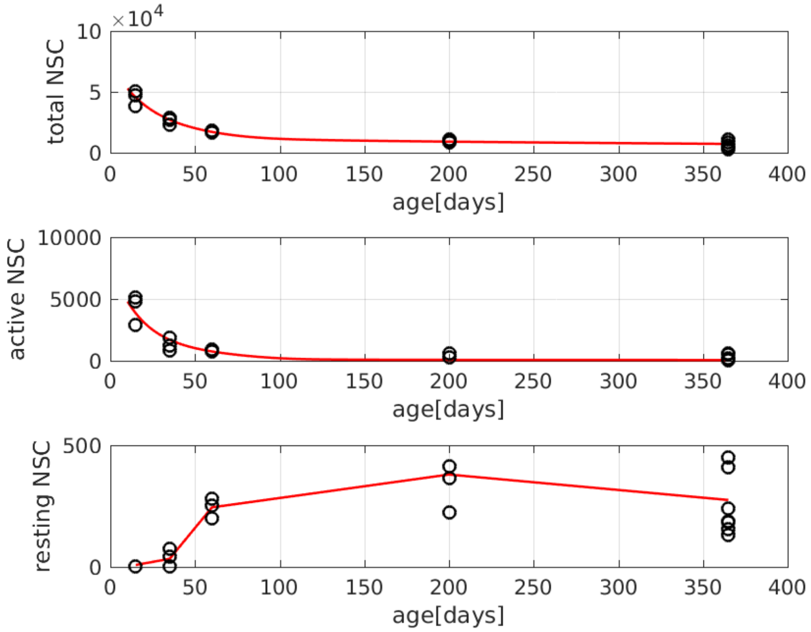

Parameter	Value	Source	Parameter	Value	Source
$r_{max,1}$ [1/days]	0.0323	fitted	$\beta_{r,1}$ [1/days]	0.0184	fitted
$r_{max,2}$ [1/days]	3692.0	fitted	$\beta_{r,2}$ [1/days]	0.1083	fitted
a	0.3338	fitted	ϵ [1/days]	0.0040	fitted
p [1/days]	1.0526	(Brandt et al., 2012)			

AIC_c=24.2

10.3.3 Maximal number of 50 divisions

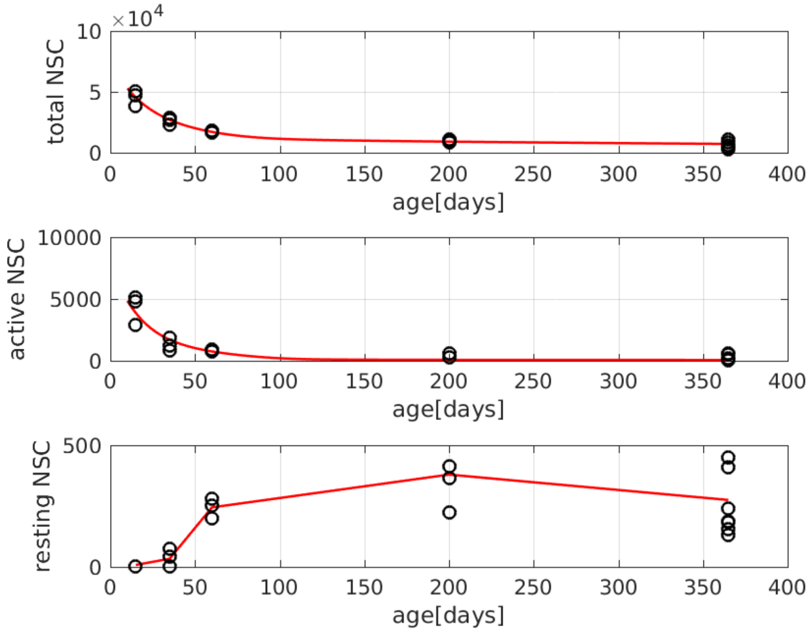

Parameter	Value	Source	Parameter	Value	Source
$r_{max,1}$ [1/days]	0.0325	fitted	$\beta_{r,1}$ [1/days]	0.0186	fitted
$r_{max,2}$ [1/days]	3692.0	fitted	$\beta_{r,2}$ [1/days]	0.1083	fitted
a	0.3322	fitted	ϵ [1/days]	0.0040	fitted
p [1/days]	1.0526	(Brandt et al., 2012)			

AIC_c=24.1

These parameters correspond to an average duration of G1-phase of 250 days in 6 month old mice.

10.4 Conclusion

We observe that AIC_c is practically identical for the different values of n, implying that there is no significant difference between the different versions of the model. Since we made a quasi steady state assumption, the unknown initial condition leads only to one additional free parameter. In the model in section 7.4.1 the initial condition led to three additional free parameters. For this reason the model considered in this section leads to a smaller AIC_c.

If we apply an analogous quasi steady state assumption to the model from section 7.4.1, i.e., $c_a^0 = -\frac{r_1 c_d^0}{(2a-1)p}$, $c_r^0 = \frac{2apc_a^0}{r_2}$, we obtain the following fit.

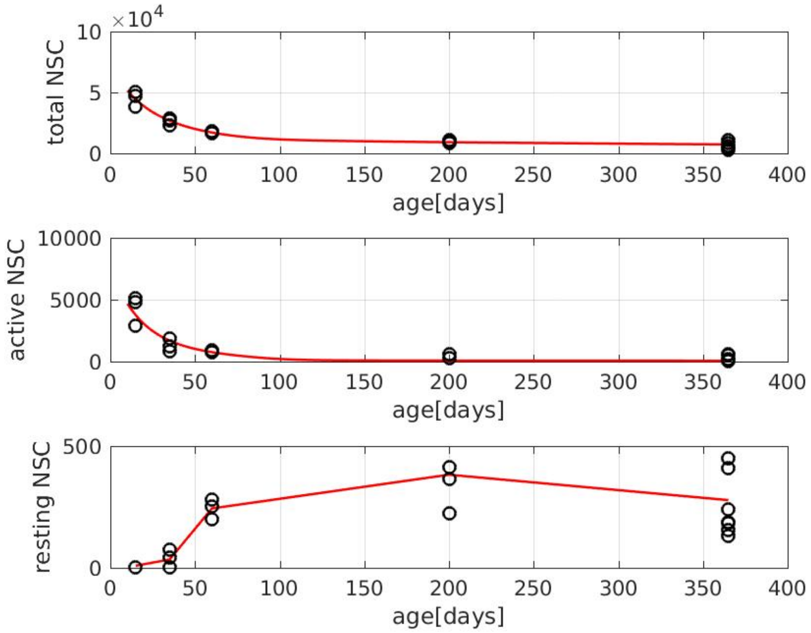

Parameter	Value	Source	Parameter	Value	Source
$r_{max,1}$ [1/days]	0.0313	fitted	$\beta_{r,1}$ [1/days]	0.0182	fitted
$r_{max,2}$ [1/days]	3390.0	fitted	$\beta_{r,2}$ [1/days]	0.1068	fitted
a	0.3357	fitted	ϵ [1/days]	0.0039	fitted
p [1/days]	1.0526	(Brandt et al., 2012)			

AIC_c=24.2

This implies that there is no significant difference between the model from section 7.4.1 with a quasi steady state assumption on initial conditions (as presented in section 10.4) assuming a return to quiescence and the models from sections 10.3.1-10.3.3 (assuming no return to quiescence). Therefore, based on the mathematical modeling we cannot distinguish between a scenario with return to quiescence and a scenario with intermittent G1 phases.

In fact both models are very similar: NSCs can switch between an actively dividing state and a resting state (either G0 or G1). The main difference between the models is that the model considered in this section limits the number of stem cell divisions before differentiation to n, whereas in the model from section 7.4.1 the distribution of the number of divisions performed by NSCs before differentiation solely depends on the value of the self-renewal fraction a. For this reason for large values of n we expect both models to have very similar properties.

References

Bonaguidi, M., Wheeler, M., Shapiro, J., Stadel, R., Sun, G., Ming, G., and Song, H. In vivo clonal analysis reveals self-renewing and multipotent adult neural stem cell characteristics. *Cell*, 145(7):1142–55, 2011.

Brandt, M., Huebner, M., and Storch, A. Brief report: Adult hippocampal precursor cells shorten s-phase and total cell cycle length during neuronal differentiation. *Stem Cells.*, 30(12):2843–7, 2012.

Burnham, K. and Anderson, D. *Model selection and multimodel inference: A practical information-theoretic approach.* Springer, 2002.

Encinas, J., Michurina, T., Peunova, N., Park, J., Tordo, J., Peterson, D., Fishell, G., Koulakov, A., and Enikolopov, G. Division-coupled astrocytic differentiation and age-related depletion of neural stem cells in the adult hippocampus. *Cell Stem Cell.*, 8(5):566–79, 2011.

Kalamakis, G., Bruene, D., Ravichandran, S., Bolz, J., Fan, W., Ziebell, F., Stiehl, T., Catala-Martinez, F., Kupke, J., Zhao, S., Llorens-Bobadilla, E., Bauer, K., Limpert, S., Berger, B., Christen, U., Schmezer, P., Mallm, J., Berninger, B., Anders, S., Del Sol, A., Marciniak-Czochra, A., and Martin-Villalba, A. Quiescence modulates stem cell maintenance and regenerative capacity in the aging brain. *Cell.*, 176(6):1407–1419, 2019.

Marciniak-Czochra, A., Stiehl, T., Ho, A., Jaeger, W., and Wagner, W. Modeling of asymmetric cell division in hematopoietic stem cells–regulation of self-renewal is essential for efficient repopulation. *Stem Cells Dev.*, 18(3):377–385, 2009.

Pilz, G., Bottes, S., Betizeau, M., Joerg, D., Carta, S., Simons, B., Helmchen, F., and Jessberger, S. Live imaging of neurogenesis in the adult mouse hippocampus. *Science.*, 359(6376):658–662, 2018.

Stiehl, T. and Marciniak-Czochra, A. Characterization of stem cells using mathematical models of multistage cell lineages. *Math Comput Modeling.*, 53(7-8):1505–1517, 2011.

Stiehl, T. and Marciniak-Czochra, A. Stem cell self-renewal in regeneration and cancer: Insights from mathematical modeling. *Curr Opinion Systems Biology.*, 5:112–120, 2017.

Ziebell, F., Dehler, S., Martin-Villalba, A., and Marciniak-Czochra, A. Revealing age-related changes of adult hippocampal neurogenesis using mathematical models. *Development.*, 145(1):dev153544, 2018.

Ziebell, F., Martin-Villalba, A., and Marciniak-Czochra, A. Mathematical modelling of adult hippocampal neurogenesis: effects of altered stem cell dynamics on cell counts and bromodeoxyuridine-labelled cells. *J R Soc Interface.*, 11(94):20140144, 2014.
